# TYMS promotes genomic instability and tumor progression in *Ink4a/Arf* null background

**DOI:** 10.1038/s41388-023-02694-7

**Published:** 2023-04-27

**Authors:** Maria V. Guijarro, Akbar Nawab, Peter Dib, Sandra Burkett, Xiaoping Luo, Michael Feely, Elham Nasri, Robert P. Seifert, Frederic J. Kaye, Maria Zajac-Kaye

**Affiliations:** 1 Department of Anatomy and Cell Biology, University of Florida College of Medicine, Gainesville, FL 32610 USA; 2 Molecular Cytogenetics Core Facility, CCR, National Cancer Institute, NIH, Frederick, MD USA; 3 Department of Pathology, Immunology and Laboratory Medicine, University of Florida College of Medicine, Gainesville, FL 32608 USA; 4 Department of Pathology, Immunology and Laboratory Medicine, University of Florida College of Medicine, Gainesville, FL 32610 USA; 5 Department of Medicine, University of Florida College of Medicine, Gainesville, FL 32610 USA

**Keywords:** Oncogenes, Cancer models

## Abstract

We previously showed that elevated TYMS exhibits oncogenic properties and promotes tumorigenesis after a long latency, suggesting cooperation with sequential somatic mutations. Here we report the cooperation of ectopic expression of human TYMS with loss of *Ink4a/Arf*, one of the most commonly mutated somatic events in human cancer. Using an *hTS/Ink4a/Arf* ^*−/−*^ genetically engineered mouse model we showed that deregulated TYMS expression in *Ink4a/Arf* null background accelerates tumorigenesis and metastasis. In addition, tumors from TYMS-expressing mice were associated with a phenotype of genomic instability including enhanced double strand DNA damage, aneuploidy and loss of G1/S checkpoint. Downregulation of TYMS in vitro decreased cell proliferation and sensitized tumor cells to antimetabolite chemotherapy. In addition, depletion of TYMS in vivo by TYMS shRNA reduced tumor incidence, delayed tumor progression and prolonged survival in *hTS/Ink4a/Arf* ^*−/−*^ mice. Our data shows that activation of TYMS in *Ink4a/Arf* null background enhances uncontrolled cell proliferation and tumor growth, supporting the development of new agents and strategies targeting TYMS to delay tumorigenesis and prolong survival.

## Introduction

TYMS is a folate-dependent essential enzyme that generates the sole intracellular de novo source of dTMP, required for DNA synthesis and repair [1]. In addition, elevated levels of TYMS mRNA and protein have been associated with worse prognosis for a wide range of hematological and solid tumors [2–4]. For example, there are >900 publications in PubMed that link either anti-cancer drug resistance or worse clinical outcome in patients with tumors expressing elevated TYMS levels [1, 4–7]. There are >100 publications in PubMed that report resistance to pemetrexed in lung cancers associated with elevated TYMS levels or sustained benefit from pemetrexed in lung cancers associated with reduced TYMS levels [1]. Evidence that TYMS can directly participate in tumorigenesis was demonstrated by (i) transformation of mammalian cells in vitro following ectopic expression of human TYMS resulting in tumor formation in vivo in nude mice [8, 9] and (ii) induction of pancreatic islet cell adenoma following expression of ectopic TYMS (previously designated hTS) in transgenic mice [10].

TYMS transcription is regulated by the E2F family of transcription factors [11–13] that controls the transition from G1 to S phase of the cell cycle and its activity is negatively regulated by underphosphorylated retinoblastoma tumor suppressor (RB). Following inactivation by cyclin-mediated phosphorylation, RB undergoes a conformational change to release E2F-1 which activates the expression of TYMS and other enzymes required for DNA replication, DNA damage repair and regulation for proper entry in the S phase [11]. Thus, loss of TYMS regulation resulting in aberrant constitutive expression is predicted to deregulate DNA synthesis and disrupt intracellular dNTP balance [14].

We previously showed that overexpression of TYMS in transgenic mice induced the development of tumors after a long latency [10], suggesting that aberrantly elevated TYMS cooperates with other cancer gene mutations to drive the neoplastic process. We recently reported that TYMS overexpression cooperates with Men1 inactivation in pancreatic islet cells and demonstrated that ectopic TYMS directly accelerated pancreatic neuroendocrine progression, reducing survival of a genetically engineered *hTS/Men1*^*−/−*^ mouse model (GEMM) [15]. Here, we report for the first time that TYMS overexpression cooperates with *Ink4a/Arf* inactivation in a novel GEMM that overexpresses human TYMS.

*lNK4A/ARF* locus is one of the most commonly mutated loci in human cancer and encodes two overlapping tumor suppressors, p16^INK4a^ and p14^ARF^ (p19 in mouse). p16^INK4a^ is a cyclin-dependent kinase inhibitor that acts upstream of the RB protein to promote cell cycle arrest [16]. p14^ARF^ is translated from an alternative reading frame from p16^INK4a^ and activates p53 by interfering with its negative regulator *MDM2* [17]. Therefore, *INK4A/ARF* mutations can disable both the RB and p53 tumor suppressor pathways [18].

Since loss of INK4A/ARF expression is one of the most common somatic events in human tumors and is associated with elevated TYMS levels [13, 19, 20], we reasoned that activated TYMS may cooperate with *INK4a/ARF* loss to promote tumor growth. In this report we demonstrated that ectopic TYMS expressed in a heterozygote *Ink4a/Arf* ^+/-^ mice shortens survival in newly established GEMM designated *hTS*/*Ink4a/Arf* ^+/−^. In addition, we show that activated TYMS promotes tumor growth and metastases in homozygote *hTS*/*Ink4a/Arf* ^*−/−*^ GEMM and can enhance DNA damage and genomic instability in tumor cells derived from *hTS/Ink4a/Arf* ^*−/−*^ mice. We also show that TYMS inhibition in vitro reduces cell proliferation and sensitizes tumor cells to antimetabolite chemotherapy currently used in clinic. In addition, we demonstrate that TYMS inhibition in vivo by TYMS shRNA prolongs survival and reduces tumor incidence in *hTS/Ink4a/Arf* ^*−/−*^ mice.

## Results

### Ectopic TYMS enhances tumor growth and shortens survival in heterozygous *Ink4a/Arf* mice

**Fig. 1:**
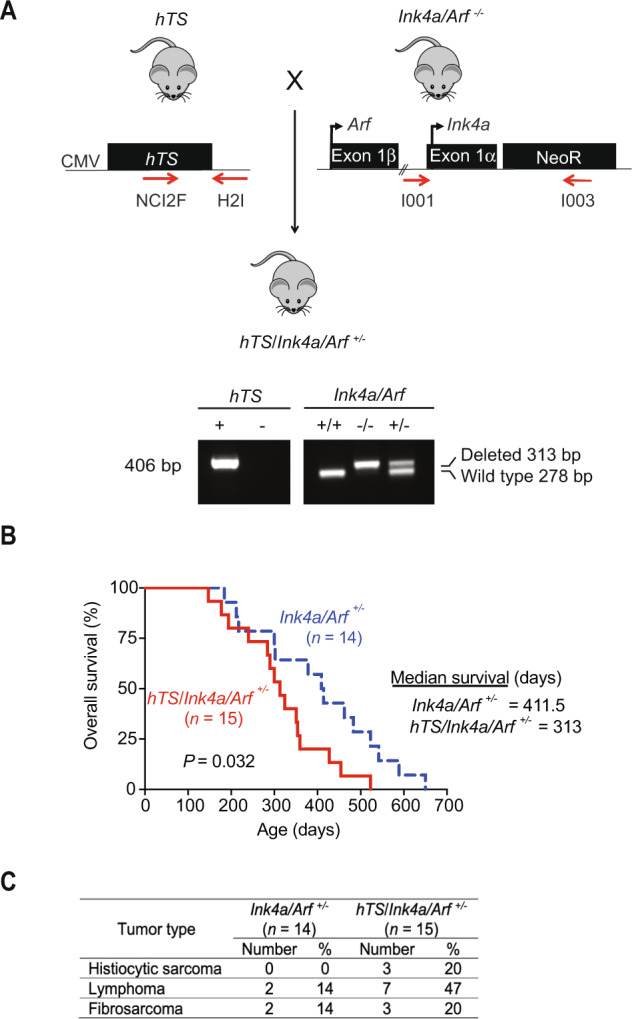
Ectopic human TYMS (hTS) enhances tumor growth and shortens survival in heterozygous *Ink4a/Arf* mice. **A** Breeding scheme to generate *hTS*/*Ink4a/Arf* ^*+/*–^ mice by crossing hTS transgenic mice with *Ink4a/Arf* ^*−/−*^ mice. Location of forward and reverse primers for the detection of hTS transgene and *Ink4a/Arf* locus are depicted by arrows. Detection of the wild-type allele was performed as described in Methods. Representative PCR analysis of gDNA extracted from mice tails shows presence (+) or absence (–) of 406 bp hTS band or wild-type 278 bp or deleted 318 bp *Ink4a/Arf* bands. CMV cytomegalovirus promoter, NeoR neomycin resistance cassette. **B** Ectopic hTS decreases survival in heterozygous *Ink4a/Arf* ^*+/–*^ mice. Kaplan-Meir survival analysis of control *Ink4a/Arf* ^*+/–*^ (broken line, *n* = 14) and hTS/*Ink4a/Arf* ^*+/*–^ (solid line, *n* = 15) mice. *hTS*/*Ink4a/Arf* ^*+/–*^ median survival was 313 days (95% CI, 240–359 days) compared to 411.5 days (95% CI, 217–542 days) in *Ink4a/Arf* ^*+/–*^ mice (*P = 0.032 by log-rank Mantel-Cox test). **C** Incidence of histiocytic sarcoma, lymphoma and fibrosarcoma is increased by ectopic hTS. Number and percentage of mice with tumors in *Ink4a/Arf* ^*+/–*^ and *hTS*/*Ink4a/Arf* ^*+/–*^ are shown.

We previously show that ectopic expression of human thymidylate synthase (hTS or TYMS) can directly participate in tumorigenesis of murine cells resulting in tumor formation in nude mice [8, 9]. In addition, activated TYMS induced pancreatic islet cell adenoma development in a mouse model although a prolonged latency (ranging from 9 to 24 months of age) was required [10]. Therefore, we reasoned that activated TYMS may cooperate with other somatic genetic lesions to enhance tumorigenesis. To test this hypothesis, we chose the Ink4a/Arf deletion since it is one of the most frequent alterations in human tumors. Hence, we crossed mice overexpressing hTS [10] with *Ink4a/Arf* null mice [21] to generate *hTS/Ink4a/Arf* ^*+/–*^ mice on a mixed FVB/129/Sv background (Fig. 1A). Genotyping for hTS transgene (406 bp) and *Ink4a/Arf* locus (313 bp for deleted allele and 278 bp for *Ink4a* wildtype allele) was determined by PCR as described in Methods (Fig. 1A). We compared survival between *hTS/Ink4a/Arf* ^*+/–*^ and *Ink4a/Arf* ^*+/-*^ mice and observed a statistically significant decrease in survival in *hTS/Ink4a/Arf* ^*+/–*^ (*n* = 15) compared to *Ink4a/Arf* ^*+/–*^ mice (*n* = 14) (*P* = 0.032, median survival 313 days for *hTS/Ink4a*/Arf ^+/–^ mice and 411.5 days for *Ink4a/Arf* ^*+/–*^ mice) (Fig. 1B). Similar to previous reports [21], *Ink4a/Arf* ^*+/−*^ mice developed lymphoma and fibrosarcoma and we found that overexpression of TYMS resulted in a higher incidence of these tumors in *hTS/Ink4a/Arf* ^*+/-*^ mice (Fig. 1C). For example, 7 out of 15 *hTS/Ink4a/Arf* ^*+/–*^ mice developed lymphoma as compared to 2 out of 14 *Ink4a/Arf* ^*+/–*^ mice (47% vs 14% respectively) (Fig. 1C). We did not observe a significant increase in fibrosarcoma (3/15 (20%) *hTS/Ink4a/Arf* ^*+/–*^ vs 2/14 (14%) in *Ink4a/Arf* ^*+/–*^ mice) but 3 out of 15 *hTS/Ink4a/Arf* ^*+/–*^ mice (20%) developed histiocytic sarcoma while this tumor was not observed in *Ink4a/Arf* ^*+/-*^ mice (Fig. 1C). To test if TYMS overexpression was involved in senescence in vivo, we extracted RNA from frozen tumors samples and performed qPCR for the senescence markers and SA β-Gal staining in freshly harvested tumors. We did not observe significant differences between genotypes (Figs. S1 and S2), suggesting that TYMS does not induce a senescent phenotype in vivo. These data show that high TYMS levels does not induce senescence in vivo and shortens overall survival due to accelerated tumor progression with more extensive infiltration in different organs in *Ink4a/Arf* ^*+/−*^ mice.

### Ectopic TYMS enhances tumor infiltration and increases incidence of haemopoietic neoplasms in *hTS/Ink4a/Arf*^*−/−*^ mice

We next tested if TYMS overexpression plays a role in tumor development in homozygous *Ink4a/Arf* ^*−/−*^ mice. At the time of death all organs were collected from *hTS/Ink4a/Arf* ^*−/−*^ (*n* = 84) and *Ink4a/Arf* ^*−/−*^ (*n* = 52) mice and tissues were analyzed by a masked pathologist. We observed that overexpression of TYMS was associated with enhanced widespread involvement in lymphomas and histiocytic sarcomas in *hTS/Ink4a/Arf* ^*−/−*^ as compared to *Ink4a/Arf* ^*−/−*^ mice (Table 1). In addition, we observed an increased incidence of histiocytic sarcoma (31.0% vs 15.4%, *P* = 0.042) and soft tissue sarcomas, excluding fibrosarcomas (5.9% vs 0%, *P* = 0.0487) in *hTS/Ink4a/Arf* ^*−/−*^ as compared to control *Ink4a/Arf* ^*−/−*^ mice (Table 2). We also observed that the incidence of histiocytic sarcoma was significantly higher in *hTS/Ink4a/Arf* ^*−/−*^ males as compared to males from the control *Ink4a/Arf* ^*−/−*^ group (42.1% vs 8.3%, *P* = 0.00045) (Table 2). Histiocytic sarcoma is a rare myeloid-derived neoplasm, more common in males than females and defined as a histiocytic or macrophage tumor (also designated as histiocytic lymphoma or malignant histiocytosis) [22, 23]. Its main histological feature is the presence of macrophages infiltrating into the organs [24]. Interestingly, of the 26 *hTS/Ink4a/Arf* ^*−/−*^ mice that developed histiocytic sarcoma, 22 (85%) showed tumor infiltration in multiple tissues ranging from 4 to 9 organs (Table 1) compared to *Ink4a/Arf* ^*−/−*^ mice where histiocytic sarcoma predominantly localized to lymph nodes and spleen (Table 1). This data shows a 3.4-fold increase in tumor infiltration of histiocytic sarcoma in TYMS expressing mice compared to control mice (*P* = 0.0034) (Table 1).

**Table 1:** Hematopoietic tumor invasion in *hTS*/*Ink4a/Arf* ^*−/−*^ vs. *Ink4a/Arf* ^*−/−*^.

Tumor type	*Ink4a/Arf* ^*−/−*^		*hTS*/*Ink4a/Arf* ^*−/−*^	
	number/Total mice	%	number/Total mice	%
Histiocytic Sarcoma				
Lesion restricted in 1–3 organs^a^	6/8	75	4/26	15
Lesion invasion to in 4–9 organs^b^	2/8	25	22/26	85**
Lymphoma				
Lesion restricted in 1–3 organs^a^	29/38	76	29/53	55
Lesion invasion to in 4–9 organs^b^	9/38	24	24/53	45*

^a^Lymph node, spleen and liver;

^b^Lymph node, spleen, liver, kidney, bone marrow, pancreas, stomach, lung, small intestine, skeletal muscle, brain, spine, thymus, heart, ovary, uterus, testes, gallbladder, soft tissue, adrenal gland and skin.

**P < 0.01, *P < 0.05.

**Table 2:** Tumor incidence in *Ink4a/Arf* ^*−/−*^ and *hTS/Ink4a/Arf* ^*−/−*^ mice.

Tumor type	*Ink4a/Arf* ^*−/−*^ (*n* = 52)		*hTS/Ink4a/Arf* ^*−/−*^ (*n* = 84)	
	mice with tumor/total mice	%	mice with tumor/total mice	%
Histiocytic Sarcoma	8/52	15.4	26/84	31.0*
Male	2/24	8.3	16/38	42.1**
Female	6/28	21.4	10/46	21.7
Lymphoma	38/52	73.1	53/84	63.1
Male	17/24	71.8	20/38	52.6
Female	21/28	75.0	33/46	71.7
Fibrosarcoma	10/52	19.2	16/84	19.0
Male	8/24	33.3	9/38	23.7
Female	2/28	7.1	7/46	15.2
Soft tissue sarcoma^a^ and teratoma	0/52	0	6/84	7.14*

^a^Non-fibrosarcoma with tumor mass bigger than 1g: liposarcoma (*n* = 1), rhabdomyosarcoma (*n* = 2), leiomyosarcoma (*n* = 1), endometrial stromal sarcoma (*n* = 1).

*P < 0.05; **P < 0.005; ***P < 0.001.

**Fig. 2:**
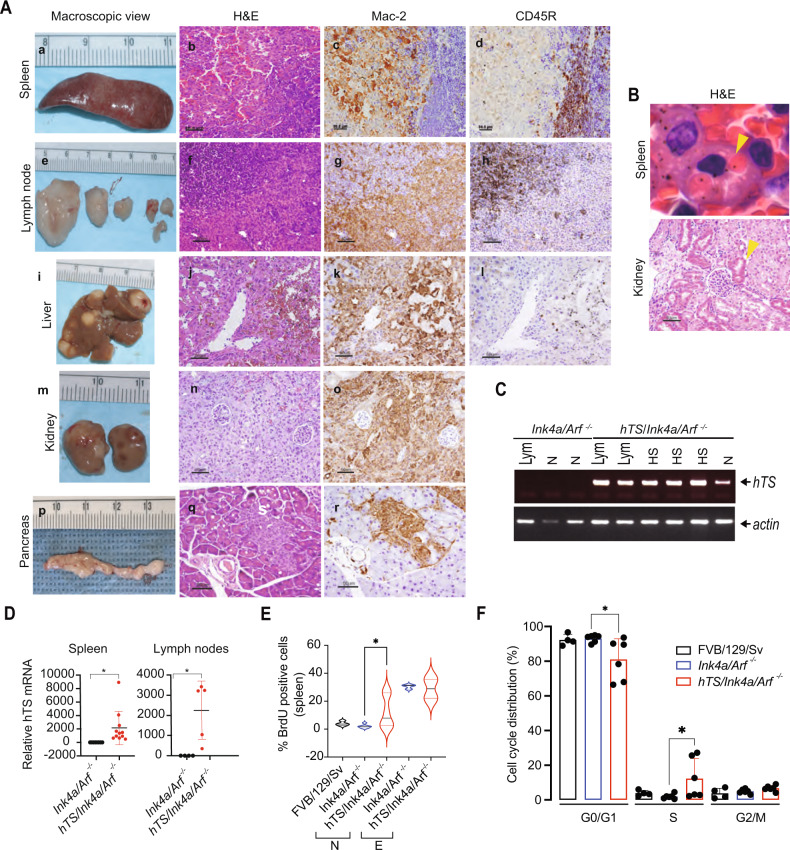
Ectopic TYMS (hTS) enhances tumor infiltration and increases incidence of histiocytic sarcoma in *hTS*/*Ink4a/Arf*^−/−^ mice. **A** Histiocytic sarcoma infiltrating multiple organs in *hTS/Ink4a/Arf* ^*−/−*^ mice. Macroscopic spleen (mouse ID 5219), lymph nodes (mouse ID 5213), liver (mouse ID 5219), kidney (mouse ID 5242), and pancreas (mouse ID 5172) images, histological evaluation by H&E stained FFPE sections and immunostaining for Mac-2 and CD45R are shown at survival endpoint. Mac-2-positive and CD45R-negative staining discriminates histiocytic sarcoma cells from lymphoma in spleen and lymph nodes. Scale bar represents 80 μm in spleen and 50 μm in sections from other organs. **B** Histological analysis showing H&E stained FFPE sections of spleen and kidney from mice diagnosed with histiocytic sarcoma. Upper panel, erythrophagocytosis (yellow arrowhead) in splenic macrophages. Lower panel, hyaline droplet nephropathy (black arrowhead) in kidney infiltrated with histiocytic sarcoma. Scale bar represents 50 μm. **C** hTS mRNA expression detected by RT-PCR analysis in spleens isolated from *hTS/Ink4a/Arf* ^*−/−*^ and *Ink4a/Arf* ^*−/−*^ mice affected with lymphoma (Lym) or histiocytic sarcoma (HS) as compared to normal (N) spleen. Actin is used as loading control. **D** hTS mRNA is upregulated in spleens and lymph nodes of tumor bearing *hTS/Ink4a/Arf* ^*−/−*^ as compared to *Ink4a/Arf* ^*−/−*^ mice. RT-qPCR showing relative TS mRNA levels in *Ink4a/Arf* ^*−/−*^ (*n* = 8) and *hTS/Ink4a/Arf* ^*−/−*^ (*n* = 11) spleens (left panel, *P = 0.0264) and *Ink4a/Arf* ^*−/−*^ (*n* = 4) and *hTS/Ink4a/Arf* ^*−/−*^ (*n* = 5) lymph nodes (right panel, *P = 0.0180). Values represent mean ± SD. **E** hTS overexpression increases ratio of splenic cells in S-phase. Violin plots showing the proportion of BrdU^+^ cells in splenic single cell suspensions from 5.9 months old mice IP injected with 1 mg of BrdU 4 h before sacrifice. Control (FVB/129/Sv, *n* = 4), *Ink4a/Arf* ^*−/−*^ (*n* = 8) and *hTS/Ink4a/Arf* ^*−/−*^ (*n* = 8) mice with normal size (N) and enlarged spleen (E) are shown. Values represent median ± quartiles. * *P* = 0.0152 by Mann-Whitney test. **F** Proportion of cells (in %) in G0/G1, S and G2/M phases of cell cycle for control background (FVB/129/Sv, *n* = 4), *Ink4a/Arf* ^*−/−*^ (*n* = 6) and *hTS/Ink4a/Arf* ^*−/−*^ (*n* = 6) mice with normal spleen. Values represent mean ± SD. * *P* = 0.0152 by Mann-Whitney test.

*hTS*/*Ink4a/Arf* ^*−/−*^ histiocytic sarcomas presented as nodules on the surface of the spleen, kidney, lymph node, liver and occasionally pancreas (Fig. 2A- a, e, i, m, p). Tumor cells were large, with abundant eosinophilic cytoplasm and pleomorphic nuclei with large nucleoli (Fig. 2A- b, f, j, n, q). In addition, 2 typical features of these tumors were found: erythrophagocytosis in spleen macrophages (which shows macrophages engulfing red blood cells) (Fig. 2B- upper panel), and hyaline droplet nephropathy in the kidney (Fig. 2B- lower panel). To confirm the diagnosis of histiocytic sarcoma, we performed differential immunophenotyping by immunohistochemistry of the tumors and observed positive expression for the mouse macrophage markers Mac-2 (Figs. 2A- c, g k, o, r and S3C) and CD11b as determined by flow cytometry (Fig. S3). No expression of B lymphocyte marker-CD45R (Figs. 2A- d, h, l and S3C) or T lymphocyte marker-CD3 (Fig. S3) were found. We also observed that 2% of *hTS/Ink4a/Arf* ^*−/−*^ mice spleens and lymph nodes affected with lymphoma also presented histiocytic sarcoma (Fig. 2A- c, d and lymph nodes in 2A- g, h) as defined by mutually exclusive staining of CD45R and Mac-2 (Fig. S3C).

In addition to the analysis of histiocytic sarcoma, we also compared lymphomas in *hTS/Ink4a/Arf* ^*−/−*^ to control *Ink4a/Arf* ^*−/−*^ mice (Tables 1 and 2). Overexpression of TYMS in *Ink4a/Arf* ^*−/−*^ mice did not increase the incidence of lymphoma, however we observed that similarly to histiocytic sarcomas, lymphomas were more aggressive with extensive multiorgan infiltration. Of the 53 *hTS/Ink4a/Arf* ^*−/−*^ mice that developed lymphoma, 24 showed tumor infiltration from 4 to 9 organs (45%) while lymphoma infiltration in *Ink4a/Arf* ^*−/−*^ mice was predominantly localized in lymph nodes and spleen (24%) (Table 1). This data shows 1.9-fold increase in tumor infiltration of lymphomas in TYMS expressing mice compared to control mice (*P* = 0.0467).

To confirm TYMS overexpression in tumors compared to normal tissues, total RNA was isolated from normal spleen and tumor tissues from *hTS/Ink4a/Arf* ^*−/−*^ and *Ink4a/Arf* ^*−/−*^ mice and presence of TYMS was determined by RT-PCR (Fig. 2C). Spleens infiltrated with lymphoma or histiocytic sarcoma (*n* = 5) showed higher TYMS expression levels as compared to normal spleens (*n* = 1) from *hTS/Ink4a/Arf* ^*−/−*^ mice (Fig. 2C). No TYMS expression was detected in normal spleen or in a spleen affected with lymphoma from *Ink4a/Arf* ^*−/−*^ mice (Fig. 2C). In addition, quantitative RT-PCR for TYMS demonstrated a 300 to 9000-fold upregulation of TYMS expression in spleen and lymph nodes isolated from *hTS/Ink4a/Arf* ^*−/−*^ as compared to *Ink4a/Arf* ^*−/−*^ tumor bearing mice (*P* < 0.05) (Fig. 2D). Therefore, TYMS overexpression in an *Ink4a/Arf* null background increases the incidence of histiocytic sarcoma and can increase tumor infiltration of both histiocytic sarcomas and lymphomas compared to *Ink4a/Arf* ^*−/−*^ control (Tables 1 and 2). These data suggest that transgenic TYMS accelerates the progression and infiltration of hematopoietic neoplasms in *Ink4a/Arf* ^*−/−*^ mice.

To investigate if TYMS overexpression was involved in accelerating tumor growth by altering the deregulated cell cycle kinetics, we measured DNA content and incorporation of the thymidine analog bromodeoxyuridine (BrdU) into splenic cells of 5.9-month-old mice in vivo (Fig. 2E, F). *hTS/Ink4a/Arf* ^*−/−*^ (*n* = 8), *Ink4a/Arf* ^*−/−*^ (*n* = 9) and control FVB/129/Sv (*n* = 4) mice were injected intraperitoneally (IP) with 1 mg BrdU. After 4 h, mice were euthanized and single-cell suspensions of splenocytes were fixed, stained with anti-BrdU antibody and DNA incorporation was measured by flow cytometry. The proportion of cells in S phase (BrdU^+^) in normal spleen of *hTS*/*Ink4a/Arf* ^*−/−*^ mice was 6-fold higher (12.28%, *n* = 6) than in normal spleen of *Ink4a/Arf* ^*−/−*^ mice (1.95%, *n* = 6, *P* = 0.0152) and 3-fold higher than in control FVB/129/Sv mice (3.91%, *n* = 4) (Fig. 2E). In contrast, when spleens were enlarged, we did not observe a significant difference in the proportion of cells in S-phase between *hTS*/*Ink4a/Arf* ^*−/−*^ and *Ink4a/Arf* ^*−/−*^ mice (28.86% vs 30.44%, *n* = 2 and 3, respectively), indicating that TYMS accelerates early tumor progression. In addition, the proportion of cells in G0/G1 phase in normal spleens was significantly lower in *hTS*/*Ink4a/Arf* ^*−/−*^ (81.01%) as compared to *Ink4a/Arf* ^*−/−*^ (93.11%, *P* = 0.0152) or control FVB/129/Sv mice (92.27%) whereas the proportion of cells in G2/M did not show any significant differences between both genotypes compared to control mice (6.71% in *hTS*/*Ink4a/Arf* ^*−/−*^, 4.94% in *Ink4a/Arf* ^*−/−*^ and 3.82% in control mice) (Fig. 2F). Thus, TYMS overexpression in an *Ink4a/Arf* ^*−/−*^ background may be promoting S-phase entry inducing cells to undergo cell division and enhance proliferation.

Since we showed enhancement of hematopoietic neoplasia following overexpression of TYMS in *Ink4a/Arf* ^*−/−*^ mice we investigated whether human lymphomas also expressed high TYMS levels. Comparison of TYMS expression across human cancer types from the TCGA dataset analyzed using the UALCAN portal [25] revealed that TYMS is expressed at higher levels in all tumor types compared to normal tissues (Fig. S4A). In addition, diffuse large B-cell lymphoma (DLBCL) samples from the same dataset were included in the group of tumors with high TYMS expression (Fig. S4B). To confirm this finding and to compare TYMS levels among different non-Hodgkin lymphoma transcriptomes, we used a different dataset [26] that showed B and T cell lymphomas (*n* = 32) express high TYMS levels (Fig. S4C) that correlated with low levels of CDKN2A expression (Fig. S4D). These data suggest that TYMS upregulation is a common event in both solid tumors and also in human non-Hodgkin´s lymphomas and may cooperate with the loss of *CDKN2A* to accelerate tumor growth and invasion, as we observed in our *hTS*/*Ink4a/Arf* ^*−/−*^ mice model.

### Ectopic TYMS induces soft tissue sarcomas in *Ink4a/Arf* null mice

**Fig. 3:**
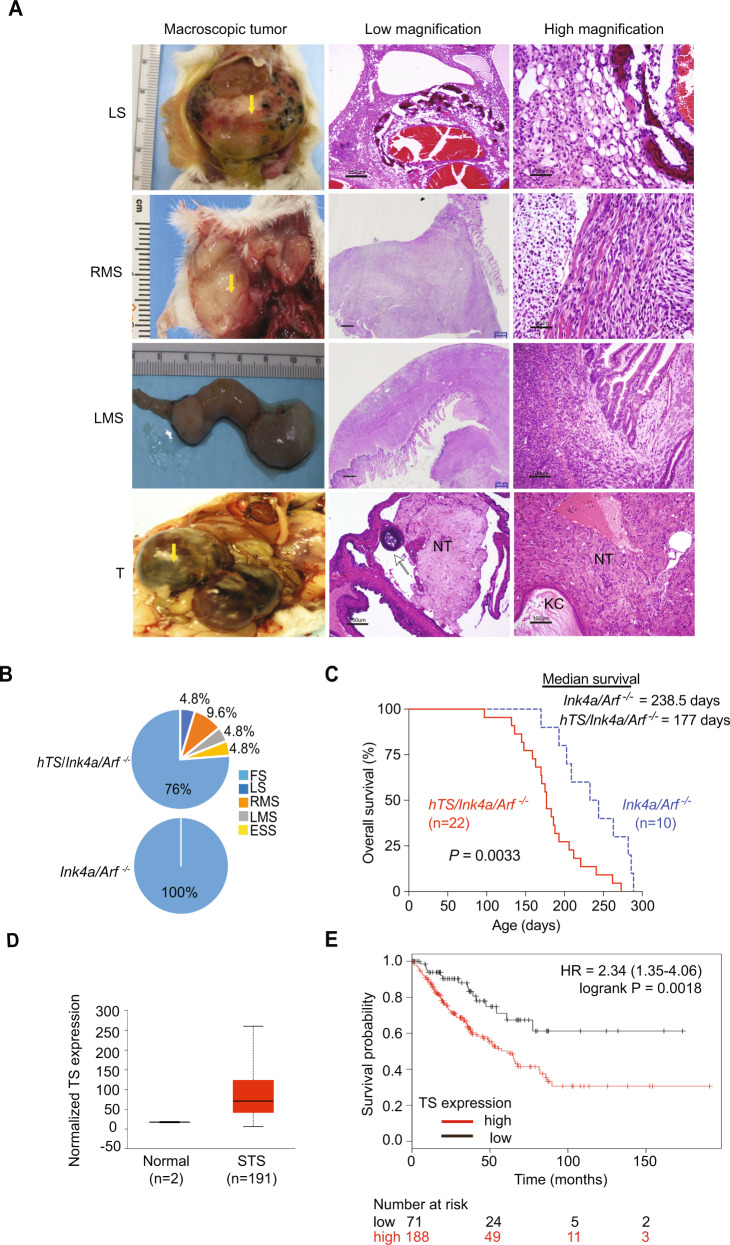
Ectopic TYMS (hTS) induces soft tissue sarcomas in *Ink4a/Arf* null mice. **A** hTS induces development of soft tissue sarcomas (STS). Left panel, representative macroscopic images of murine *hTS/Ink4a/Arf* ^*−/−*^ soft tissue sarcomas; black arrow points to the tumor mass. Middle and right column: low and high magnification of H&E-stained FFPE sections showing characteristic features from soft tissue sarcoma. LS: liposarcoma (scale bar represents 250 μm) with typical spindle shaped neoplastic cells visible at higher magnification (scale bar represents 50 μm). RMS: rhabdomyosarcoma (scale bar represents 600 μm) showing spindle cells arising from preexisting skeletal muscle at higher magnification (scale bar represents 50 μm). LMS: leiomyosarcoma (scale bar, 600 μm); spindle cells at higher magnification (scale bar represents 100 μm). T: Teratoma with an ovarian follicle (black arrow) and neural tissue (NT) (scale bar, 250 μm) and a keratin cyst (KC) at higher magnification (scale bar represents 100 μm). **B** Ectopic hTS increases the incidence of liposarcoma (LS), rhabdomyosarcoma (RMS), leiomyosarcoma (LMS) and endometrial stromal sarcoma (ESS). Pie charts showing percentage of each type of soft tissue sarcoma in *hTS/Ink4a/Arf* ^*−/−*^ mice (*n* = 22) compared to *Ink4a/Arf* ^*−/−*^ mice (*n* = 10). FS: Fibrosarcoma. **C** Ectopic hTS significantly decreases survival in *hTS/Ink4a/Arf* ^*−/−*^ compared to *Ink4a/Arf* ^*−/−*^. Kaplan Meier survival analysis of *hTS/Ink4a/Arf* ^*−/−*^ (solid red line, *n* = 22) and *Ink4a/Arf* ^*−/−*^ (broken blue line, *n* = 10) mice. Median survival for *hTS/Ink4a/Arf* ^*−/−*^ mice was 177 days (95% CI, 159–206 days,) as compared to 238.5 days (95% CI, 193–286 days) for *Ink4a/Arf* ^*−/−*^ mice (**P = 0.0033, by log-rank Mantel-Cox test). Only fibrosarcomas were observed in the control *Ink4a/Arf* ^*−/−*^ group. **D** TS is highly expressed in human STS (*n* = 191) compared to normal tissues (*n* = 2). TS expression measured as transcripts per million (TPM). Results shown are based upon data generated by the TCGA Research Network: https://www.cancer.gov/tcga and analyzed using UALCAN portal. **E** High expression of TS correlates with poor survival in patients diagnosed with STS. Kaplan-Meier survival curves based on TS expression in human STS patients. Red: High TS expression. Black: low TS expression. TCGA soft tissue sarcoma RNA-seq dataset (excluding MPST) was analyzed in the R 2.15.0 statistical environment. Hazard ratio (HR) and log-rank *P* values were calculated. The “survplot” R package is applied for generating the Kaplan-Meier plot.

In addition to lymphoma and histiocytic sarcoma, *Ink4a/Arf* ^*−/−*^ mice also developed fibrosarcoma (19.2%) (Table 2). TYMS expression in these mice did not increase the overall incidence of fibrosarcoma, although we observed 2-fold higher fibrosarcoma incidence in *hTS*/*Ink4a/Arf* ^*−/−*^ females compared to control *Ink4a/Arf* ^*−/−*^ females (15.2% vs 7.1% respectively). However, statistical significance was not reached perhaps due to low frequency of fibrosarcoma in female mice (Table 2). We also observed that TYMS overexpression induced soft tissue sarcoma (STS) and ovarian teratoma that were not detected in *Ink4a/Arf* ^*−/−*^ mice (7.14 % vs 0%, *P* = 0.0487) (Table 2 and Fig. 3A). *hTS*/*Ink4a/Arf* ^*−/−*^ mice developed large STS such as liposarcoma (LS), rhabdomyosarcomas (RMS) and leiomyosarcoma (LMS) that ranged from 1.5 g to 10.1 g. Representative histology is shown in Fig. 3A. LS showed with typical lobules of neoplastic fat cells, cavernous blood vessels and foci of mineralization with vacuolated and spindle shaped neoplastic cells (Fig. 3A). RMS were located in the subcutis beneath the dermis, with neoplastic cells extending from the muscularis externa into the mucosa and spindle cells arising from preexisting skeletal muscle (Fig. 3A). LMS showed neoplastic spindle cells extending from muscularis externa into the mucosa in the stomach (Fig. 3A). As shown in Fig. 3b, 4.8% of *hTS/Ink4a/Arf* ^*−/−*^ with STS were diagnosed with LS, endometrial stromal sarcoma (ESS) or LMS and 9.6% with RMS. Interestingly, 80% of *hTS/Ink4a/Arf* ^*−/−*^ animals that developed STS (except LMS) were also diagnosed with lymphoma and fibrosarcoma or histiocytic sarcoma (Table S1). In addition, 50% of *hTS/Ink4a/Arf* ^*−/−*^ mice that developed fibrosarcoma were also diagnosed with other tumors such as lymphoma or histiocytic sarcoma. In contrast, only 3 out of 10 *Ink4a/Arf* ^*−/−*^ mice (30%) with fibrosarcoma were diagnosed with lymphoma or histiocytic sarcoma (Table S1). As expected, *hTS/Ink4a/Arf* ^*−/−*^ mice did not develop pancreatic neuroendocrine tumors (PanNET) as shown previously in *hTS* transgenic mice [10]. *hTS/Ink4a/Arf* ^*−/−*^ mice had a shorter survival time (6–9 months) due to lymphoma and sarcoma compared to *hTS* mice that developed PanNET at low frequency and required a latency period of approximately 9–12 months of age. Therefore, *Ink4a/Arf* null mice (that are prone to develop soft tissue sarcoma) exposed to an oncogenic stimulus such as TYMS overexpression, develop not only several subtypes of soft tissue sarcoma but also lymphomas and histiocytic sarcomas in the same animal.

To further study the effect of TYMS overexpression in mice that developed soft tissue sarcoma, we compared survival of *hTS/Ink4a/Arf* ^*−/−*^ mice diagnosed with soft tissue sarcoma including fibrosarcoma vs *Ink4a/Arf* ^*−/−*^ mice that only developed fibrosarcoma. *hTS/Ink4a/Arf* ^*−/−*^ mice (*n* = 22) had a statistically significant decrease in overall survival as compared to *Ink4a/Arf* ^*−/−*^ mice (*n* = 10), (*P* = 0.0033, median survival 177 days vs 238.5 days, respectively) (Fig. 3C). In summary, our data suggests that TYMS expression results in more aggressive phenotype with higher incidence of hematological and mesenchymal tumors in the same mouse. This data also suggests that TYMS expression may be contributing to more rapid progression of soft tissue sarcoma, apart from fibrosarcoma, in the *Ink4a/Arf* ^*−/−*^ mice that are already predisposed to soft tissue sarcoma development.

Since we observed that TYMS overexpression in *Ink4a/Arf* ^*−/−*^ mice increased the incidence of soft tissue sarcoma and significantly decreased *hTS/Ink4a/Arf* ^*−/−*^ survival compared to *Ink4a/Arf* ^*−/−*^ mice, we analyzed TYMS levels in human soft tissue sarcoma. Data from The Cancer Genome Atlas (TCGA) [27] reveled that soft tissue sarcoma was among the group of tumors with highest levels of TYMS (Fig. S4) and TYMS transcripts were 3-fold more abundant in soft tissue sarcoma (*n* = 191) than in normal tissues (*n* = 2) (Fig. 3D). We also observed that patients with low TYMS mRNA had significantly longer survival than the group of patients with high TYMS mRNA levels (*P* = 0.0018) (Fig. 3E), suggesting that TYMS may play oncogenic role in soft tissue sarcoma progression and thus, blocking TYMS expression may increase survival in patients with soft tissue sarcoma. In summary, both human and mice data support deregulated TYMS as an oncogenic stimulus that concurrent with *Ink4a/Arf* inactivation accelerates tumor progression and decreases survival.

### TYMS downregulation reduces cell proliferation and sensitizes cells to antimetabolite therapy

**Fig. 4:**
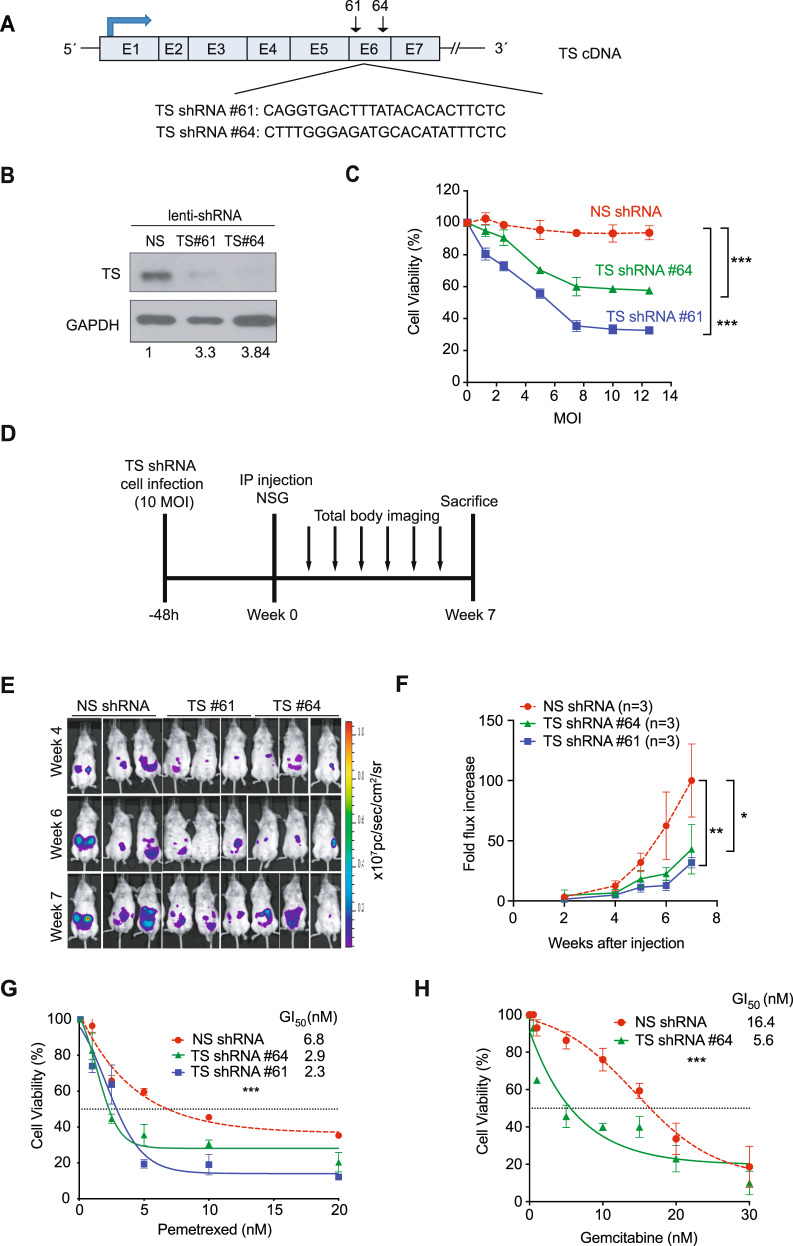
Downregulation of TYMS (TS) reduces cell proliferation and sensitizes cells to antimetabolite therapy. **A** TS cDNA showing exons 1–7 and target sequences for TS shRNA #61 and #64. **B** Western blot analysis of TS protein expression in whole-cell extracts of Luc-hTS5278 cells collected 24 h after infection with 10 MOI lentiviral shRNA: nonspecific (NS), TS shRNA#61 and #64. GAPDH is used as a loading control. Numbers denote relative TS expression levels compared to loading control by densitometric analysis. **C** TS shRNA decreases tumor cell viability. MTS viability assay of Luc-hTS5278 histiocytic sarcoma cells after 72 h transduction with lentiviral TS shRNA #61 and #64 at increasing MOI (0–12) compared to non-specific shRNA control (NS). Results are representative of 3 replicates per group and time point. Data represent mean ± SD. Statistical analysis was done using a 2-way ANOVA. ***P < 0.0001. **D** Timeline indicating experimental design to study the effect of TS downregulation in vitro. Luc-hTS5278 cells were infected with 10 MOI TS shRNA#61 and TS shRNA #64 or non-specific (NS) shRNA control. 48 h hours after, cells were injected IP into NSG mice. Mice underwent luciferase imaging weekly and sacrificed 7 weeks after injection. **E** Luc-hTS5278 cells transduced with lentiviral-TS shRNA #64 and #61 or NS shRNA and injected into NSG mice. Pictures correspond to luciferase (Luc) bioluminescence intensity at weeks 4, 5 and 7 after injection. **F** Quantification of the luciferase signal in animals injected with Luc-hTS5278 cells transduced with lentiviral TS shRNA #64 (*n* = 3), TS shRNA #61 (*n* = 3) compared to animals injected with Luc-hTS5278 cells transduced with NS shRNA (*n* = 3) over time. Data represents mean ± SEM. * *P* = 0.0263, **P = 0.0026, calculated by 2-way ANOVA. **G** TS downregulation sensitizes cells to pemetrexed. Viability assay of Luc-hTS5278 cells stably transfected with lentiviral TS shRNA#61 and #64 treated with pemetrexed (1–20 nM) for 72 h compared to NS shRNA (non-specific control) infected cells. GI_50_ is shown. Results are representative of 3 replicates per group and drug concentration. Data represent mean ± SD. ***P < 0.0001, calculated by 2-way ANOVA. **H** TS downregulation sensitizes cells to gemcitabine. Viability assay of Luc-hTS-5278 cells stably transfected with lentiviral TS shRNA#64 treated with gemcitabine (1–30 nM) for 72 h compared to NS shRNA (non-specific control) infected cells. GI_50_ is shown. Results are representative of 3 replicates per group and drug concentration. Data represent mean ± SD. ***P < 0.0001, calculated by 2-way ANOVA.

To directly test if high TYMS levels is responsible for the increased tumor proliferation in *hTS/Ink4a/Arf* ^*−/−*^ mice, we asked whether TYMS downregulation would result in decreased cell proliferation. We established a cell line derived from a *hTS/Ink4a/Arf* ^*−/−*^ splenic tumor, designated hTS5278 (see detail description in Material and Methods) that was further infected with the luciferase reporter to track cells in vivo (Luc-hTS5278). Luc-hTS5278 cells were infected with lentiviral TS shRNAs designated #61 and #64 or non-specific control (NS shRNA). TS shRNA #61 targeted the 5´ region of exon 6 and TS shRNA #64 the 3´ region of the same exon (Fig. 4A). Knockdown of TYMS in Luc-hTS5278 cells infected with either 10 multiplicities of infection (MOI) lentiviral-TS shRNA #61 or #64 was confirmed by immunoblot analysis while infection with NS shRNA had no effect on TYMS levels following 24 h treatment (Fig. 4B). To obtain the optimal titer of lentiviral particles to inhibit cell proliferation by silencing TYMS expression we used increasing doses of both lentiviral TS shRNAs and NS shRNA (2 to 12 MOI) and performed MTS assay 72 h after infection. Cell viability decreased in a dose-dependent manner in Luc-hTS5278 cells infected with either TS shRNA #61 or #64 compared to NS shRNA control infected cells (*P* < 0.0001) (Fig. 4C). At 10 MOI Luc-hTS5278 cells viability decreased between 40 and 60% with TS shRNA #64 and #61 respectively and higher MOI did not further decreased proliferation. Thus, we established that 10 MOI of shRNA was required to achieve the highest decrease in hTS5278 cellular viability in vitro (Fig. 4C). These data showed that TS shRNAs #61 and #64 efficiently decrease TYMS levels and suggest that TYMS downregulation is sufficient to reduce proliferation in *hTS/Ink4a/Arf* ^*−/−*^ derived tumor cells.

To test the effect of TYMS downregulation on tumor growth in vivo, Luc-hTS5278 cells transduced with 10 MOI TS shRNA #61, #64 or NS shRNA were injected IP into immunocompromised NSG mice (*n* = 3 per group). Tumors were monitored by bioluminescence imaging once a week for 7 weeks (Fig. 4D). At 5 weeks after injection a reduction of luciferase was observed in animals injected with Luc-hTS5278 transduced with either TS shRNA #61 or #64 compared to mice injected with NS shRNA (Fig. 4E, F). At weeks 6 and 7, luciferase signal consistently increased in control animals injected with NS shRNA (ranged between 2 and 5-fold increase) compared to animals injected with either TS shRNA #61 or #64 (*P* = 0.0026; *P* = 0.0263, respectively) (Fig. 4E, F). These data suggest that TYMS knockdown in tumor cells derived from *hTS/Ink4a/Arf* ^*−/−*^ mice delays tumor onset and tumor cell growth in NSG mice.

To study whether reduction of TYMS level would sensitize tumor cells to the antimetabolite chemotherapy agents pemetrexed or gemcitabine, we transiently transduced Luc-hTS5278 cells with lentiviral 10 MOI TS shRNA #61, #64 or NS shRNA and then treated cells with increasing concentrations of either drug. Pemetrexed is a multitargeted antifolate that can be easily administered to patients and it shows antitumor activity in a wide range of tumor types whereas gemcitabine does not directly interact with TYMS. Hence, we hypothesized that if we reduce TYMS levels, we would sensitize tumor cells to respond to lower doses of pemetrexed or gemcitabine that were otherwise ineffective in the presence of elevated TYMS commonly detected in clinical patient samples. Following 24 h incubation with TS shRNA, cells were treated with increasing concentrations of either pemetrexed or gemcitabine, then Luc-hTS5278 cell viability was compared to cells infected with NS shRNA. We observed that cells with low TYMS levels were more sensitive to pemetrexed treatment as shown by reduced cell viability with a decrease in the GI_50_ (TS shRNA #61 GI_50_ = 2.3 and TS shRNA #64 GI_50_ = 2.9 nM compared to NS shRNA infected cells with GI_50_ = 6.8 nM, *P* < 0.0001) (Fig. 4G). Similarly, when Luc-hTS5278 cells with downregulated TYMS (shRNA #64) were treated with increasing concentrations of gemcitabine, the GI_50_ decreased to 5.6 nM compared to 16.4 nM of control cells (*P* < 0.0001) (Fig. 4H). In summary, these data show that downregulation of TYMS expression with shRNA inhibits hematopoietic tumor growth in vivo and suggests that lowering TYMS levels enhanced tumor cell inhibition in vitro by pemetrexed or gemcitabine treatment.

### Ectopic TYMS induces double strand DNA damage and enhances genomic instability in tumor cells derived from *hTS*/*Ink4a/Arf*^*−/−*^ mice

**Fig. 5:**
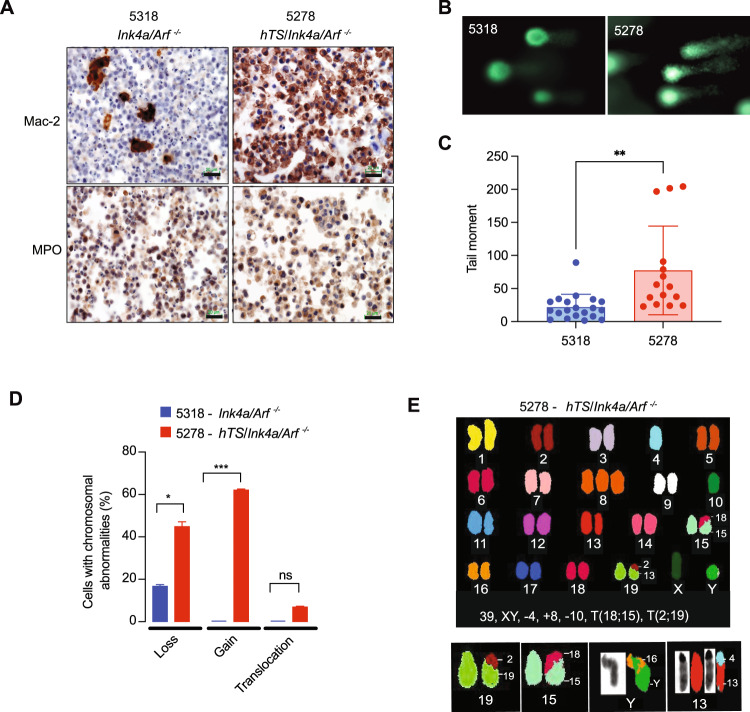
Overexpression of TYMS (hTS) induces double strand DNA damage and enhances genomic instability in tumor cells derived from *hTS/Ink4a/Arf*^−/−^ GEMM. **A** hTS5278 and 5318 derived tumor cell lines are of histiocytic/monocytic origin. Representative immunohistochemistry with Mac-2 and myeloperoxidase (MPO) in 5318 and hTS5278 histiocytic sarcoma cell lines derived from tumors isolated from *Ink4a/Arf* ^*−/−*^ and *hTS/Ink4a/Arf* ^*−/−*^ mice respectively. Scale bar represents 20 μm. **B** Comet assay demonstrating double strand breaks in cells overexpressing TYMS. Representative images of 5318 and hTS5278 cell nuclei stained with SYBR green after electrophoresis at neutral pH and observed under the fluorescent microscope. Longer tail comets can be visualized in hTS5278 cells. **C** Quantification of comet tail lengths comparing 5318 (*n* = 20) and hTS5278 cell nuclei (*n* = 15). Data represents mean ± SD. Statistical significance was determined by unpaired two tailed t-test, **P = 0.0012. **D** Ectopic hTS induces chromosomal rearrangements. Upper panel, representative image of SKY karyotype of one hTS-5278 metaphase spread. Number of chromosomes and karyotype is annotated, T: translocation. Lower panel, chromosomal translocations from 2 different hTS5278 metaphases detected by SKY analysis: T(2;19) and T(18;15) were detected together in the same metaphase spread. T(Y;16) and T(4;13) were found together in another metaphase spread. G-band staining for chromosomes Y and 13 is also shown. Numbers next to the aberrant chromosomes indicate the origin of the translocated material. **E** Ectopic hTS increases chromosomal abnormalities. Percentage of loss, gain and translocation detected by spectral karyotyping (SKY) in histiocytic sarcoma derived cells. Karyotypes from 5318 control cells (*n* = 24) and hTS5278 cells (*n* = 29) were quantified. Data represents mean ± SD. *P = 0.0399, *** *P* < 0.0001 by two-sided Fisher´s exact test, ns: not significant.

It has been reported that impaired nucleotide biosynthesis and nucleotide imbalance would enhance the rate of DNA replication errors, DNA damage and ultimately genomic instability [28]. Thus, we reasoned that overexpression of TYMS, by dysregulating cellular dNTP pools [29], may enhance DNA damage and increase genomic instability. We first examined whether high TYMS levels would cause DNA double strand breaks (DSB) in primary tumor cell lines derived from *hTS/Ink4a/Arf* ^*−/−*^ and *Ink4a/Arf* ^*−/−*^ mice. hTS5278 cells were established from a *hTS/Ink4a/Arf* ^*−/−*^ mouse spleen and 5318 cell line was derived from an *Ink4a/Arf* ^*−/−*^ mouse lymph node. Mac-2 and MPO immunostaining, markers of histiocytic/monocytic origin, are shown for both cell lines (Fig. 5A). Using hTS5278 and 5318 cells we performed a neutral comet assay to detect double strand breaks (DSB) by measuring the capacity of negatively charged DNA fragments to be pulled through an agarose gel in response to an electric field, appearing like a comet or tail (Fig. 5B). Our data shows that TYMS overexpression increased 3-fold the mean tail moment in hTS5278 (77.36, *n* = 15) compared to 5318 control cells (21.67, *n* = 20) (Fig. 5C), confirming that overexpression of TYMS in *Ink4a/Arf* ^*−/−*^ tumor cells contributes to a statistically significant increase (*P* = 0.0012) in double strand DNA damage.

Since DSB in DNA are precursors to further genome damage that may result in chromosomal aberrations [30], we asked whether high TYMS expression induced chromosomal instability (CIN). Spectral karyotyping (SKY) analysis comparing hTS5278 and control 5318 cells revealed that among the hTS5278 karyotypes (*n* = 29) the number of chromosomes varied from 37 to 41 in all single cells analyzed and only 21% (*n* = 6) single cells had normal karyotype (40 chromosomes). In contrast, karyotypic analysis of control 5318 cells (*n* = 24) showed normal karyotypes in 79% (*n* = 19) single cells and 21% (*n* = 5) with 37 to 39 chromosomes per cell (Table S2). These results suggest genetic heterogeneity and significant increase of chromosomal aberrations in TYMS overexpressing cells as compared to control cells (*P* = 0.0471). hTS5278 showed higher percentage of chromosomal losses compared to 5318 cells (44.8% vs 16.7%, *P* = 0.0399 respectively), and chromosomal gains (62.1%) and translocations (6.9%) were only found in TYMS expressing cells (Fig. 5D and Table S2). In addition, only in hTS5278 cells we recurrently found a trisomy of chromosome 8 in 17 out of 29 metaphases (Fig. 5E) and four chromosomal translocations [T(2;19), T(18;15), T(Y;16) and T(4;13)] (2 translocations per metaphase in 2 out of 29 metaphases). Thus, elevated TS levels in an *Ink4a/Arf* ^*−/−*^ background induces further chromosomal rearrangements and aneuploidy. These data suggest that TYMS expression in histiocytic/monocytic hematopoietic cells lacking *Ink4a/Arf* induces double strand DNA damage that leads to the acquisition of structural and numerical chromosomal changes and genomic instability that may be a contributing factor to enhancement of tumor progression in *hTS/Ink4a/Arf* ^*−/−*^ mice.

### Downregulation of human TYMS prolongs survival of *hTS/Ink4a/Arf*^*−/−*^ mice

**Fig. 6:**
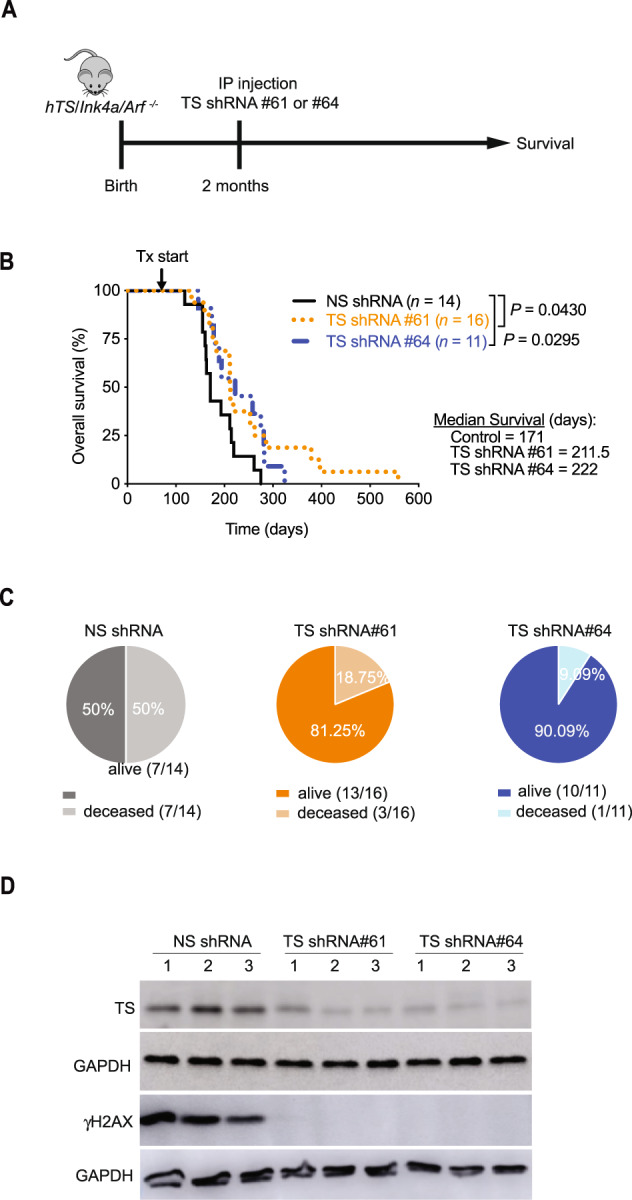
Downregulation of human TYMS (hTS) increases survival of *hTS/Ink4a/Arf*^−/−^ GEMM. **A** Experimental design showing timeline to study the effect of TS downregulation in vivo. *hTS/Ink4a/Arf* ^*−/−*^ mice were injected with one dose of lentiviral TS shRNA#61 and TS shRNA #64 or non-specific (NS) shRNA control at 2 months of age and sacrificed when showed signs of sickness at the end of life. **B** Kaplan-Meier survival graph of *hTS/Ink4a/Arf* ^*−/−*^ mice after one IP injection of TS shRNA#61 (*n* = 16) and TS shRNA#64 (*n* = 11) compared to control non-specific shRNA (NS, *n* = 14). Median survival for animals injected with TS shRNA#61 was 211.5 days (95% CI, 176–286 days,) for shRNA#64 group was 222 days (95% CI, 174–282 days) and for NS shRNA group was 171 days (95% CI, 155–219). *P = 0.0430 and *P = 0.0295, respectively calculated by log-rank Mantel-Cox test. **C** Percent of live and deceased animals treated with TS shRNA#61 and #64 compared to NS shRNA at 171 days. This time point corresponds to the median survival for NS shRNA control group. **D** Splenic tumors derived from *hTS/Ink4a/Arf* ^*−/−*^ treated with TS shRNA #61 and #64 compared to control NS shRNA immunoblotted for TS and γH2AX (Ser139). Three representative tumors per group are shown. GAPDH is used as loading control.

Since TYMS downregulation reduced hTS5278 cell proliferation in vitro and delayed tumor growth in NSG mice in vivo (Fig. 4), we asked whether TS shRNA treatment prolongs survival of *hTS/Ink4a/Arf* ^*−/−*^ mice. Two-month-old mice were injected IP with lentiviral-TS shRNA #61 (*n* = 16) and TS shRNA #64 (*n* = 11) and survival was compared to control *hTS/Ink4a/Arf* ^*−/−*^ mice injected with non-specific (NS) shRNA (*n* = 14) (scheme in Fig. 6A). TYMS downregulation significantly increased median survival in both TS shRNAs treated mice as compared to NS shRNA control (TS shRNA #61: 211.5 days and TS shRNA #64: 222 days compared to NS shRNA: 171 days, *P* = 0.0430 and *P* = 0.0295, respectively) (Fig. 6B). When we compared the number of *hTS/Ink4a/Arf* ^*−/−*^ mice alive at 171 days, we observed that 81% and 90% of TS shRNA #61 and #64 treated mice respectively, remained alive compared to 50% of NS shRNA treated mice (Fig. 6C). Immunoblot analysis of spleens (3 mice per group, age between 173 and 282 days) showed that TYMS levels in tumors from *hTS/Ink4a/Arf* ^*−/−*^ mice treated with lentiviral-TS shRNA #61 and TS shRNA #64 were 100% reduced at endpoint, compared to animals injected with lentiviral NS shRNA (Fig. 6D). Therefore, our results show that one dose of TS shRNA injection into *hTS/Ink4a/Arf* ^*−/−*^ mice was sufficient to reduce TYMS levels during mice lifetime and the lower TYMS expression levels was associated with a significant prolongation of mice survival as compared to NS shRNA treated controls.

Since we observed induction of DNA damage *in* hTS5278 cells as compared to 5318 cells using comet assay (Fig. 5B, C), we asked whether TYMS downregulation would decrease DNA damage in vivo. We tested spleen tissues using immunoblot analysis to measure steady-state levels of histone H2A variant H2AX phosphorylated at Ser139 (γH2AX), a molecular marker of DNA damage. We observed negligible γH2AX expression levels in *hTS/Ink4a/Arf* ^*−/−*^ mice injected with lentiviral-TS shRNA #61 or TS shRNA #64 as compared to mice treated with lentiviral-NS shRNA (Fig. 6D). These data indicate that downregulation of TYMS expression was associated with a reduction of γH2AX, further suggesting that TYMS overexpression plays a role in accelerating tumor progression and downregulation of TYMS levels reduces DNA damage and prolongs survival of *hTS/Ink4a/Arf* ^*−/−*^ mice.

## Discussion

Although TYMS has been studied for many years as a tumor prognostic marker and therapeutic target, we previously reported that ectopic overexpression of human TYMS (also designated hTS) can transform cells in vitro and result in tumor formation in nude mice [8, 31]. In addition, we developed transgenic hTS mice and demonstrated that ectopic TYMS overexpression promoted tumorigenesis but only after a long latency [10]. This prolonged latency suggested that aberrant TYMS expression cooperates with other stochastic somatic events to enhance neoplastic progression and metastasis. To test this hypothesis, we have now studied the ability of ectopic TYMS to cooperate with *Ink4a/Arf* deletion in a defined mouse tumor model to promote tumorigenesis. In this report we show that TYMS overexpression in *Ink4a/Arf* null background accelerates tumor progression, enhances tumor metastases, decreases overall survival and induces DNA damage and genomic instability. Importantly, we observed that TYMS cooperates with heterozygote *Ink4a/Arf* inactivation increasing the underlying tumor incidence and decreasing survival of *hTS/Ink4a/Arf* ^*+/-*^ mice that otherwise show indolent tumors with a long latency. In addition, we showed that ectopic TYMS markedly increased tumor growth and metastasis in *hTS/Ink4a/Arf* ^*−/−*^ mice. We observed a significant increase in the incidence of histiocytic sarcomas at earlier age and a marked increase in histiocytic sarcoma and lymphoma dissemination to multiple organs in *hTS/Ink4a/Arf* ^*−/−*^ compared to *Ink4a/Arf* ^*−/−*^ mice. In addition, we observed a significant increase in the incidence of soft tissue sarcomas and reduced survival in *hTS*/*Ink4a/Arf* ^*−/−*^ compared to *Ink4a/Arf* ^*−/−*^ mice. These observations provide biological context to clinical studies that have shown that elevated TYMS levels correlate with poor clinical outcome [32, 33]. Therefore, instead of high TYMS levels arising as a secondary response to increase tumor growth rates, we observed that high TYMS levels may be directly driving the increase in tumor growth, tissue invasiveness and metastasis [5].

To further study the oncogenic effects of ectopic TYMS, we examined the impact on cell cycle kinetics in spleen tissues from *hTS/Ink4a/Arf* ^*-/-*^ mice. We showed that ectopic TYMS significantly increased the number of cells in S-phase, implying that ectopic TYMS enhanced *Ink4a/Arf* ^*−/−*^ cells to enter S-phase. Loss of *Ink4a* and *Arf* genes have been previously associated with S-phase entry and activation of DNA damage checkpoints [34]. Our data now show that loss of *Ink4a/Arf* concurrent with ectopic TYMS expression further increases the number of cells in S-phase and DNA DSB in *hTS/Ink4a/Arf* ^*−/−*^ tumor-derived cells. Moreover, our data demonstrate that tumor cells derived from *hTS/Ink4a/Arf* ^*−/−*^ mice exhibited elevated levels of chromosomal gains, losses and translocations compared to tumor cells derived from *Ink4a/Arf* ^*−/−*^ mice. In addition, TYMS regulates methyl group transfers that plays a role in nucleotide biosynthesis and folate-mediated one-carbon metabolism and thus dysregulation of these pathways by high levels of TYMS may result in aberrant DNA replication [35, 36], increased DNA damage, chromosomal instability and cancer. However, a direct connection linking these DNA instability events with imbalance in nucleotide and folate cellular pools remains to be defined. Overall, these data suggest that loss of *Ink4a/Arf* gene function may cooperate with ectopic aberrant TYMS levels to accelerate genomic instability that would promote cancer development and metastases.

A role for deregulated TYMS to enhance cell proliferation of cells derived from *hTS/Ink4a/Arf* ^*−/−*^ tumor was further tested by downregulating TYMS protein using shRNA. We observed reduced cell viability in a dose-dependent manner in tumor cells treated with lentiviral TS shRNA. Moreover, tumor onset and progression were delayed in NSG mice injected with tumor cells derived from *hTS/Ink4a/Arf* ^*−/−*^ mice, that were transduced with lentiviral TS shRNA compared to control animals injected with cells carrying a non-specific shRNA. Further confirmation of the effect of TYMS knockdown was performed in *hTS/Ink4a/Arf* ^*−/−*^ mice. Lentiviral TS shRNA IP injection resulted in a prolonged survival of *hTS/Ink4a/Arf* ^*−/−*^ mice that was associated with a potent and sustained TYMS silencing and a reduction in DNA damage as shown by decrease in γH2AX levels. This data shows a direct correlation between decreased TYMS levels, delayed tumor progression and enhanced survival as important clinical implications.

In addition, TYMS inhibition sensitized tumor cells derived from *hTS/Ink4a/Arf* ^*−/−*^ mice to pemetrexed and gemcitabine treatment further supporting the clinical impact of regulating TYMS levels. Previous studies have shown that overexpression of TYMS in human non-small cell lung cancer (NSCLC) tumor cell lines reduced sensitivity to pemetrexed while downregulation of TYMS restored sensitivity of these cells to the proapoptotic effect of the drug [37]. Combination therapy of TYMS shRNA and pemetrexed also sensitized mesothelioma xenografts prolonging mouse survival [38], suggesting that the clinical benefit of pemetrexed as a first-line treatment for patients with malignant pleural mesothelioma and NSCLC could improve by downregulating TYMS. It has been previously hypothesized that increased sensitivity to antifolates, such as pemetrexed, or to pyrimidine antagonists, such as gemcitabine, that is observed after TYMS knockdown is due to an imbalance between dUTP and dTTP pools with a subsequent decrease in the efficiency of DNA synthesis [39]. Furthermore, dUTP-dTTP imbalance results in misincorporation of dUTP into DNA and consequent DNA damage [39]. Our data also corroborates prior studies [37, 38] showing that downregulation of TYMS expression resulted in enhanced antiproliferative effect of pemetrexed or gemcitabine exposure. Therefore, combination of pemetrexed and gemcitabine that was tested in several clinical phase II trials [40–42] may benefit, when used in the presence of reduced TYMS levels. Since sustained antimetabolite therapy results in drug resistance that limits clinical benefit due to feedback induction of elevated TYMS levels [1] these data provide a rationale for the identification of a new class of TYMS inhibitors with a strategy to block TYMS feedback induction to avoid drug resistance.

## Material and methods

### Mice

*hTS* [10] and *Ink4a/Arf* ^*−/−*^ mice ([21]- NCI mouse repository, strain 01XB2) were previously described. To generate *hTS* transgenic mice in a FVB background, a 2540 bp NruI/NsiI fragment containing hTS cDNA driven by CMV promoter was excised from pcDNA3.1zeo-TS vector [8]. Ectopic hTS was proven to be catalytically active in mice tumor tissues retaining the ability to form a ternary complex with FdUMP [10]. Once *hTS* mice were crossed with *Ink4a/Arf* ^*−/−*^, mice were maintained on a mixed FVB/129/sv background. NSG (NOD.Cg-Prkdc^scid^ Il2rgt^m1Wjl^/SzJ strain) were generated at University of Florida Animal facility. Mice were maintained within the University of Florida Cancer Genetics Research Center and Communicore barrier facilities in individual ventilated cages. All animal experiments were done in accordance with approved protocols from the Institutional Animal Care and Use Committees (IACUC), according to national and institutional guidelines. Kaplan-Meier survival curves were compared using the log-rank Mantel-Cox test calculated in GraphPad Prism 9 (GraphPad Software, San Diego, CA, USA). Tumors and organs were systematically collected as frozen for molecular analysis and a piece of tumor/organ was fixed in 10% neutral-buffered formalin for pathology and immunohistochemical analyses.

#### Genotyping of hTS/Ink4a/Arf ^−/−^ mice

Genotype analysis in tail snips was performed by standard PCR analysis using human TS-specific primers [10] as well as a set of primer pairs designed to score for the wild-type, heterozygous, or homozygous *Ink4a/Arf* genotype. The sequences for *TYMS* (*hTS)* primers are: NCI2 5ʹ-ATGCCCTCTGCCAGTTCTATGTGG-3ʹ and H2I 5ʹ-TAGAAGGCACAGTCGAGG-3ʹ; and *Ink4a/Arf* locus primers are as follows: #I001, 5ʹ-GTGATCCCTCTACTTTTTCTTC-3ʹ, #I002, 5ʹ-CGGAACGCAAATATCGCAC-3ʹ, and #I003, 5ʹ-GAGACTAGTGAGACGTGCTAC-3ʹ. I001/I003 detects a 313 bp band for the knockout and I001/I002 a 278 bp band for the wild type.

#### Histopathology and immunohistochemistry

For routine histological analysis, specimens were fixed in 10% neutral-buffered formalin for 48 h and transferred to 70% ethanol, processed and embedded in paraffin (Histoserv Inc., Germantown, MD, USA or UF Molecular Pathology Core, Gainesville, FL). Tissues were serially sectioned (5 μm thick) and stained either by conventional H&E or with specific antibodies. H&E-stained slides were interpreted by a certified mouse pathologist (Dr. Victoria Hoffmann, Division of Veterinary Resources, Bethesda, MD; Dr. Mary K Reinhard, ACS Assistant Director at the University of Florida (UF); Dr. Elham Nasri (Bone and Soft Tissue Pathology and Clinical Assistant Professor), Dr. Robert P. Seifert (Hematopathology Program Director and Clinical Assistant Professor) and Michael Feely Gastrointestinal/liver and genitourinary Pathology and Clinical Assistant Professor) at Anatomic Pathology Department at UF, Gainesville, FL. For immunohistochemistry, sections were cut by the NCI-Frederick Pathology Lab (PHL, Frederick, MD, USA) and Molecular Pathology Core (UF, Gainesville, FL).

To prepare HistoGels, 5 × 10^6^ cells were fixed in 10% neutral-buffered formalin for 1 h, then washed twice with PBS by centrifuging at 1000 rpm for 5 min. Then cells were processed using HistoGel^TM^ (Thermofisher) according to manufacturer instructions.

Paraffin-embedded tissues or cells were then deparaffinized and prepared for staining. Antigen retrieval was performed using heated citrate buffer for 15 min, and tissues were then incubated for 30 min with primary antibodies against Mac-2 (1:1000, Cedarlane Laboratories, USA), CD45R (1:200, PharMingen), CD3 (1:100, DAKO), myeloperoxidase (1:750 DAKO). Primary antibodies were visualized with peroxidase ABC kits (Vector Labs) according to manufacturer’s instructions. Tumor cell attribution and diagnosis of tumors was performed by certified pathologist using differential immunophenotyping.

### Flow cytometry

10^6^ cells obtained from spleen or lymph nodes were blocked using rat anti–mouse CD16/CD32 Fc Block (BD Biosciences) and then stained in PBS with 2% FBS containing the BD Bioscience antibody CD11b-FITC (1.2 μg/ml). Cells were incubated on ice for 45 min. Following incubation, cell suspensions were subjected to two wash cycles involving resuspension of cell pellets in 3 mL PBS with 2% FBS, followed by centrifuging at 1000 rpm at 4 °C for 5 min, followed by supernatant removal. Finally, samples were resuspended in 300 mL of PBS with 2% FBS and analyzed by flow cytometry using a BD LSRII FACScan flow cytometer and analyzed using the BD FACSDiva software.

### shRNA knockdown in *hTS/Ink4a/Arf*^*−/−*^ mice

For shRNA treatment, 2-month-old *hTS/Ink4a/Arf* ^*−/−*^ animals were injected IP with 10 MOI TYMS shRNA#61, #64 or with NS shRNA. Animals were monitored until survival endpoint. Kaplan-Meier survival curves were compared using the log-rank Mantel-Cox test calculated in GraphPad Prism 9 (GraphPad Software, San Diego, CA, USA). Tumors and organs were systematically collected and frozen for molecular analysis and a section of tumor/organ was fixed in 10% neutral-buffered formalin for pathology and immunohistochemical analyses.

### Mouse spleen and lymph node single cell suspensions

Spleens were extracted and divided longitudinally in two halves. One piece was fixed in 10% buffered formalin for histological analysis and the other piece was homogenized in PBS using the plunger of an insulin syringe, disrupting the tissue against a 100-μm nylon cell strainer. Cells were then centrifuged at 1000 rpm for 5 min to discard supernatant. Cells were washed again with PBS and counted in a Nexcelom cell counter (Perking Elmer, MA, USA).

### BrdU detection in splenic mouse cells

For BrdU labeling, 5.9-month-old *hTS/Ink4a/Arf* ^*−/−*^ animals were injected IP with 1 mg of BrdU (BD Pharmigen) and let them rest for 4 h before sacrifice to harvest spleens. Spleens were harvested and processed to obtain single cell suspensions as described above. Then, cells were stained with BrdU for flow cytometry detection. Briefly, 10^6^ cells were fixed with BD Cytofix/Cytoperm buffer for 30 min on ice, washed with BD Perm/Wash buffer and incubated in Cytoperm Plus Buffer for 10 min on ice. Cells were then re-fixed with BD Cytofix/Cytoperm buffer for 5 min at room temperature and after washed with BD Perm/Wash buffer, cells were treated with 30 μg of DNase for 1 h at 37 °C to expose incorporated BrdU. After washing thoroughly with BD Perm/Wash buffer, cells were incubated with anti-BrdU for 20 min at room temperature and prepared in staining buffer to be analyzed by flow cytometry in a BD Accuri C6 Plus cytometer (BD Biosciences, NJ, USA). Analysis was performed using FlowJo v9 (BD Biosciences, NJ, USA).

### Establishment of cell lines

Single-cell suspensions from a *hTS/Ink4a/Arf* ^*−/−*^ spleen (hTS5278 – number refers to mouse ID) and an *Ink4a/Arf* ^*−/−*^ lymph node (5318 – number refers to mouse ID) were made in RPMI 1640 (Sigma) supplemented with 10% FBS (Gibco), 1 mM sodium pyruvate (Gibco), 10 mM HEPES (Gibco), 50 μm ß–Mercaptoethanol (Sigma) and 1% Penicillin/Streptomycin (Gibco). A piece of the tissue was homogenized in PBS using the plunger of an insulin syringe, disrupting the tissue against a 100-μm nylon cell strainer. Then, 50 cells were seeded per well in a 96-well plate. After four weeks in culture, growing tumor cells were expanded to verify their genotype and negativity for Mycoplasma (Sigma). hTS5278 was further infected with lentiviral luciferase (Addgene #19785) and maintained in puromycin 8 ug/ml; the new cell line was designated Luc-hTS5278.

### Reverse-transcription PCR

Total RNA was prepared using Trizol reagent (Invitrogen). cDNA was synthesized using SuperScript reverse transcriptase (Invitrogen) following the manufacturer’s instructions. Briefly, 5 μg of total RNA was converted to cDNA, and further used for PCR analysis. Primers used for PCR were TS-3F, 5ʹ-GGGGCAGATCCAACACAT-3ʹ and TS-4R, 5ʹ-CTCCCTTGGAAGACAGCTCTTTA-3ʹ. Actin primers, included with the cDNA synthesis kit (Invitrogen), were used to normalize the PCR products. All reactions were done for 35 cycles: (95 °C for 30 s, 60 °C for 30 s and 72 °C for 60 s).

### qRT-PCR

0.2 μg of RNA was converted to cDNA and subjected to quantitative PCR using SYBR Green TaqMan Master Mix (Applied Biosystems) according to the manufacturer’s instructions. qRT-PCR products and their dissociation curves were detected using a 7500 Fast Real-Time PCR System (Applied Biosystems, Waltham, MA) with Taqman Probes for TYMS (Hs00426591_m1) and R18S (Hs99999901_s1) (Applied Biosystems). TS mRNA expression was normalized relative to that of GAPDH mRNA in the same sample.

### shRNA knockdown assays

Luc-hTS5278 cells were infected with lentiviral supernatants expressing shRNAs against TYMS (TS shRNA#61, TRCN0000045664; TS shRNA#64, TRCN0000045667; both from Sigma). A non-specific (NS) shRNA control vector (Sigma SHC002) was used as a negative control. Infected cells were seeded in 96 well plates at a density of 5 × 10^4^ cells per well and proliferation was assessed using the MTS viability assay. For in vivo studies, 4 × 10^6^ cells were infected with 10 MOI lentiviral particles for 48 h, trypsinized, and dissolved in 200 ul of PBS to be injected IP into the abdominal cavity of SCID mice. Tumor growth was measured every week using Xenogen IVIS from Perkin Elmer for 7 weeks. Animal experiments complied with University of Florida IACUC and international regulations and ethical guidelines.

### Plasmid preparation

All plasmid constructs were propagated in DH5α (Invitrogen) on LB plates or in liquid media 100 μg/ml Ampicillin at 37 °C. Liquid cultures were grown in orbital shaking at >200 RPM. Plasmid extraction was performed using maxi-prep (Qiagen) following the manufacturer’s recommendations. All constructs were verified by analytical digest and/or Sanger sequencing.

### Lentiviral production

Of, 12 × 10^6^ HEK293T were seeded per T175 flask and incubated overnight at 37 °C and 5% CO_2_. 10–20 h after seeding, HEK293T were co-transfected with lentiviral construct TS shRNA #61, TS shRNA#64 or non-specific (NS) shRNA (32 μg), viral packaging plasmid (psPAX, 16 μg) and viral envelope plasmid (pMD2.G, 16 μg) using a mixture of 1 mg/ml polyethyleneimine (Polysciences) and 1.5 M NaCl. Viral supernatant was collected, filtered through a 0.45 μm filter 40 h post-transfection and pulled together. Pooled supernatants were concentrated using an Amicon Ultra-15 100 K cutoff filter device (EMD Millipore, Burlington, MA, USA) according to manufacturer’s instructions. Final concentrated virus was aliquoted and stored at −80 °C till used. Viral titer of the concentrated supernatant was determined by a Lenti-X qRT-PCR Titration Kit (Clontech).

### Protein isolation

Protein lysates were generated using RIPA buffer (Santa Cruz) for 20 min on ice, followed by centrifugation for 15 min at 13000 rpm at 4 °C. Protein-containing supernatant was transferred to fresh microcentrifuge tubes and stored at −20 or −80 °C until further use. Protein was quantified using Bradford Assay (BioRad) following manufacturer’s recommendations, with standard curves generated with bovine serum albumin (Sigma Aldrich).

### Western blotting

In total, 20 μg of total protein lysate was loaded per lane of 10% Novex Tris Glycine Gel (Invitrogen). SDS-PAGE was run at 150 V for 1.5 to 2 hr. Proteins were transferred to nitrocellulose membranes using iBlot (Invitrogen, Waltham, MA, USA). Membranes were blocked with 5% non-fat dry milk (LabScientific) in Tris-buffered saline supplemented with Tween20 (0.1%) (TBS-T) for 45–60 min at RT. Membranes were incubated on a plate shaker overnight at 4 °C with TS-106 antibody (1:300 as previously described [8]), H2AX (Ser139) (1:1000, Cell Signaling #9718) or GAPDH (1:1000, Millipore ABS16) diluted in blocking buffer. Membranes were washed extensively with TBS-T (minimum 4X for 5 min), followed by incubation with horseradish peroxidase-conjugated secondary antibody goat anti mouse IgG (BioRad) or goat anti rabbit (BioRad) in blocking buffer 30–60 min at RT on a plate shaker. Membranes were washed extensively with TBS-T (minimum 4X for 5 min). Signal was detected using Super Signal West Pico Plus Chemiluminescent substrate (ThermoScientific) following manufacturer’s recommendations. Membranes were either developed using multiple film (Genesee Scientific, San Diego, CA) processed in a Kodak X-Omat 2000A processor with exposures ranging from 2 s to 2 min or by scanning membranes in Amersham Imager 680 for different times for optimal image analysis. For quantification of protein levels relative to loading control, densitometric analysis was performed by ImageJ software (U. S. National Institutes of Health, Bethesda, Maryland, USA).

### Cell viability assays

For proliferation assays, 5 × 10^4^ Luc-hTS5278 cells were plated in 96 well plates and 16–20 h after seeding, cells were infected with concentrated lentiviral TS shRNAs at different multiplicities of infection (MOI) (2–12 MOI). For GI_50_ determination, Luc-hTS5278 cells were seeded at 4000 cells/well in 96-well plates. 16–20 h after seeding, cells were treated with the indicated treatment: pemetrexed (LC Labs #P-7177; 1 nM to 20 nM) or gemcitabine (LC Labs #G-4177; 1 nM to 30 nM). Cells were grown in the presence of TYMS shRNA with or without gemcitabine or pemetrexed for 72 h. Cell viability was assessed by MTS using Cell Titer 96 R Aqueous One Solution Cell Proliferation Assay Kit (Promega), following manufacturer’s recommendations. Chemiluminescent output (integration time 1000 ms) was measured on a SpectraMax M3 (Molecular devices, San Jose, CA, USA). Data were normalized to max/min and plotted in GraphPad Prism GI_50_ were estimated.

### Cytogenetic analysis

hTS5278 and 5318 cells were incubated in a T25 flask with 10 μg/ml Colcemid (Karyomax, Invitrogen) 1.5 h prior to harvest. Cells were then dissociated with 0.8 ml 0.05% trypsin/EDTA and treated with 0.075 M KCl hypotonic solution for 15 min at 37 °C to be then fixed with methanol: acetic acid (3:1). Metaphase slides were prepared from the cell harvest and let dry at 42 °C overnight. The metaphases were hybridized with a 20-color mouse SKY paint kit (ASI) according to the manufacturer’s protocol [43]. Spectral images of the hybridized metaphases were acquired using a SD301 SpectraCubeTM system (Applied Spectral Imaging Inc., Carlsbad, CA, USA) mounted on top of an epifluorescence microscope Axioplan 2 (Zeiss, Dublin, CA, USA). Images were analyzed using Spectral Imaging 4.0 acquisition software (Applied Spectral Imaging Inc., Carlsbad, CA, USA). G banding was simulated by an electronic inversion of DAPI-counterstaining.

### Comet assay

Neutral comet assay was used to detect DNA double-strand breaks and was performed based on the manufacturer’s instructions (Trevigen, Gaithersburg, MD). Briefly, hTS5278 and 5318 cells were cultured for 48 h before being collected and resuspended in low-melting agarose (R&D Systems) at a final concentration of 0.9% before spreading on 20-well Comet Slides (R&D Systems). Slides were placed at 4 °C in the dark for 10 min and then immersed in prechilled lysis solution at 4 °C for 60 min to lyse cells before washing twice with deionized H_2_O and submerging Comet Slides in prechilled 1X Neutral Electrophoresis Buffer (deionized H_2_O containing Tris Base and Sodium Acetate, pH = 9) for 30 min, at 4 °C. Electrophoresis was run for 45 min with applied voltage at 21 V and a current of approximately 300 mA for 45 min using the Comet Assay Electrophoresis System II (R&D Systems, Minneapolis, MN, USA). The electrophoresis unit and buffer were chilled to 4 °C. After excess of Neutral Electrophoresis Buffer was drained, slides were gently immersed in DNA Precipitation Solution for 30 min at room temperature. Slides were rinsed twice in deionized H_2_O, fixed in 70% ethanol for 30 min at room temperature and dried at 37 °C using a standard incubator for 15 min. DNA was stained with 40 μl 1× SYBR Gold nucleic acid gel stain (S-11494, Life Technologies) per well. Comets were visualized using Olympus BX51 fluorescence microscope (Olympus Corporation, Tokyo, Japan). Double strand breaks were quantified using the OpenComet Image-J plugin [44].

### Analysis of TYMS in the TCGA soft tissue sarcoma (STS) cohort

Anonymized STS patient level data (overall survival in days, live/dead status, and RSEMv2 expression) were downloaded, and plotted using R 2.15.0 statistical environment.

TYMS expression (TPM + 1) data for STS was extracted from RNA-seq data from TCGA Research Network https://www.cancer.gov/tcga and analyzed in UALCAN portal [25].

### Statistics

Statistical analyses were performed using the GraphPad Prism 9 (GraphPad Software, USA). Values are represented as data ± SD. Data were analyzed by 2-tailed Student’s test or 2-way ANOVA for comparison between groups. Kaplan-Meier survival curves were analyzed using the log-rank Mantel-Cox test to determine significance. For all studies, *P* values <0.05 were considered statistically significant.

## Supplementary information

Supplemental Material

## Data Availability

Datasets utilized in this study were obtained from TCGA dataset and GSE120124.

## References

[1] Wilson PM, Danenberg PV, Johnston PG, Lenz HJ, Ladner RD (2014). Standing the test of time: targeting thymidylate biosynthesis in cancer therapy. Nat Rev Clin Oncol.

[2] Krajinovic M, Costea I, Chiasson S (2002). Polymorphism of the thymidylate synthase gene and outcome of acute lymphoblastic leukaemia. Lancet.

[3] Hu YC, Komorowski RA, Graewin S, Hostetter G, Kallioniemi OP, Pitt HA (2003). Thymidylate synthase expression predicts the response to 5-fluorouracil-based adjuvant therapy in pancreatic cancer. Clin Cancer Res.

[4] Lee HS, Chen M, Kim JH, Kim WH, Ahn S, Maeng K (2014). Analysis of 320 gastroenteropancreatic neuroendocrine tumors identifies TS expression as independent biomarker for survival. Int J Cancer.

[5] Edler D, Hallstrom M, Johnston PG, Magnusson I, Ragnhammar P, Blomgren H (2000). Thymidylate synthase expression: an independent prognostic factor for local recurrence, distant metastasis, disease-free and overall survival in rectal cancer. Clin Cancer Res.

[6] Saviozzi S, Ceppi P, Novello S, Ghio P, Lo Iacono M, Borasio P (2009). Non-small cell lung cancer exhibits transcript overexpression of genes associated with homologous recombination and DNA replication pathways. Cancer Res.

[7] Bertino JR, Banerjee D (2003). Is the measurement of thymidylate synthase to determine suitability for treatment with 5-fluoropyrimidines ready for prime time?. Clin Cancer Res.

[8] Rahman L, Voeller D, Rahman M, Lipkowitz S, Allegra C, Barrett JC (2004). Thymidylate synthase as an oncogene: a novel role for an essential DNA synthesis enzyme. Cancer Cell.

[9] Voeller D, Rahman L, Zajac-Kaye M (2004). Elevated levels of thymidylate synthase linked to neoplastic transformation of mammalian cells. Cell Cycle.

[10] Chen M, Rahman L, Voeller D, Kastanos E, Yang SX, Feigenbaum L (2007). Transgenic expression of human thymidylate synthase accelerates the development of hyperplasia and tumors in the endocrine pancreas. Oncogene.

[11] DeGregori J, Kowalik T, Nevins JR (1995). Cellular targets for activation by the E2F1 transcription factor include DNA synthesis- and G1/S-regulatory genes. Mol Cell Biol.

[12] Trimarchi JM, Lees JA (2002). Sibling rivalry in the E2F family. Nat Rev Mol Cell Biol.

[13] Banerjee D, Schnieders B, Fu JZ, Adhikari D, Zhao SC, Bertino JR (1998). Role of E2F-1 in chemosensitivity. Cancer Res.

[14] Reichard P (1988). Interactions between deoxyribonucleotide and DNA synthesis. Annu Rev Biochem.

[15] Vijayakurup V, Maeng K, Lee HS, Meyer B, Burkett S, Nawab A (2022). Thymidylate synthase accelerates Men1-mediated pancreatic tumor progression and reduces survival. JCI Insight.

[16] Serrano M, Hannon GJ, Beach D (1993). A new regulatory motif in cell-cycle control causing specific inhibition of cyclin D/CDK4. Nature.

[17] Kamijo T, Weber JD, Zambetti G, Zindy F, Roussel MF, Sherr CJ (1998). Functional and physical interactions of the ARF tumor suppressor with p53 and Mdm2. Proc Natl Acad Sci USA.

[18] Sherr CJ (2001). The INK4a/ARF network in tumour suppression. Nat Rev Mol Cell Biol.

[19] Weinstein JN, Collisson EA, Mills GB, Shaw KR, Ozenberger BA, The Cancer Genome Atlas Research N (2013). The Cancer Genome Atlas Pan-Cancer analysis project. Nat Genet.

[20] Hengstschlager M, Pusch O, Hengstschlager-Ottnad E, Ambros PF, Bernaschek G, Wawra E (1996). Loss of the p16/MTS1 tumor suppressor gene causes E2F-mediated deregulation of essential enzymes of the DNA precursor metabolism. DNA Cell Biol.

[21] Serrano M, Lee H, Chin L, Cordon-Cardo C, Beach D, DePinho RA (1996). Role of the INK4a locus in tumor suppression and cell mortality. Cell.

[22] Vos JA, Abbondanzo SL, Barekman CL, Andriko JW, Miettinen M, Aguilera NS (2005). Histiocytic sarcoma: a study of five cases including the histiocyte marker CD163. Mod Pathol.

[23] Hornick JL, Jaffe ES, Fletcher CD (2004). Extranodal histiocytic sarcoma: clinicopathologic analysis of 14 cases of a rare epithelioid malignancy. Am J Surg Pathol.

[24] Emile JF, Abla O, Fraitag S, Horne A, Haroche J, Donadieu J (2016). Revised classification of histiocytoses and neoplasms of the macrophage-dendritic cell lineages. Blood.

[25] Chandrashekar DS, Bashel B, Balasubramanya SAH, Creighton CJ, Ponce-Rodriguez I, Chakravarthi B (2017). UALCAN: A Portal for Facilitating Tumor Subgroup Gene Expression and Survival Analyses. Neoplasia.

[26] Pericart S, Tosolini M, Gravelle P, Rossi C, Traverse-Glehen A, Amara N (2018). Profiling immune escape in Hodgkin’s and diffuse large B-cell lymphomas using the transcriptome and immunostaining. Cancers (Basel).

[27] The Cancer Genome Atlas Research N. (2017). Comprehensive and integrated genomic characterization of adult soft tissue sarcomas. Cell.

[28] Davidson MB, Katou Y, Keszthelyi A, Sing TL, Xia T, Ou J (2012). Endogenous DNA replication stress results in expansion of dNTP pools and a mutator phenotype. EMBO J.

[29] Mathews CK (2006). DNA precursor metabolism and genomic stability. FASEB J.

[30] van Gent DC, Hoeijmakers JH, Kanaar R (2001). Chromosomal stability and the DNA double-stranded break connection. Nat Rev Genet.

[31] Zhao Z, Shi Y, Ke F, Wei S, Gui J, Zhang Q (2008). Constitutive expression of thymidylate synthase from LCDV-C induces a transformed phenotype in fish cells. Virology.

[32] Allegra CJ, Parr AL, Wold LE, Mahoney MR, Sargent DJ, Johnston P (2002). Investigation of the prognostic and predictive value of thymidylate synthase, p53, and Ki-67 in patients with locally advanced colon cancer. J Clin Oncol.

[33] Johnston PG, Lenz HJ, Leichman CG, Danenberg KD, Allegra CJ, Danenberg PV (1995). Thymidylate synthase gene and protein expression correlate and are associated with response to 5-fluorouracil in human colorectal and gastric tumors. Cancer Res.

[34] Weinberg RA (1995). The retinoblastoma protein and cell cycle control. Cell.

[35] Kotsantis P, Petermann E, Boulton SJ (2018). Mechanisms of oncogene-induced replication stress: jigsaw falling into place. Cancer Discov.

[36] Ducker GS, Rabinowitz JD (2017). One-carbon metabolism in health and disease. Cell Metab.

[37] Takezawa K, Okamoto I, Okamoto W, Takeda M, Sakai K, Tsukioka S (2011). Thymidylate synthase as a determinant of pemetrexed sensitivity in non-small cell lung cancer. Br J Cancer.

[38] Abu Lila AS, Kato C, Fukushima M, Huang CL, Wada H, Ishida T (2016). Downregulation of thymidylate synthase by RNAi molecules enhances the antitumor effect of pemetrexed in an orthotopic malignant mesothelioma xenograft mouse model. Int J Oncol.

[39] Curtin NJ, Harris AL, Aherne GW (1991). Mechanism of cell death following thymidylate synthase inhibition: 2’-deoxyuridine-5’-triphosphate accumulation, DNA damage, and growth inhibition following exposure to CB3717 and dipyridamole. Cancer Res.

[40] Ngeow J, Toh CK (2010). The role of pemetrexed combined with gemcitabine for non-small-cell lung cancer. Curr Drug Targets.

[41] Morfouace M, Shelat A, Jacus M, Freeman BB, 3rd, Turner D, Robinson S et al. Pemetrexed and gemcitabine as combination therapy for the treatment of Group3 medulloblastoma. Cancer Cell 2014; 25: 516-29. 10.1016/j.ccr.2014.02.009 PMC3994669 24684846

[42] West HL, Wakelee HA, Perry MC, Belt RJ, Chen R, Obasaju C (2009). Gemcitabine and pemetrexed administered in rapid sequence as front-line chemotherapy for advanced non-small-cell lung cancer: a phase II clinical trial. Ann Oncol.

[43] Schrock E, du Manoir S, Veldman T, Schoell B, Wienberg J, Ferguson-Smith MA (1996). Multicolor spectral karyotyping of human chromosomes. Science.

[44] Gyori BM, Venkatachalam G, Thiagarajan PS, Hsu D, Clement MV (2014). OpenComet: an automated tool for comet assay image analysis. Redox Biol.

