# Supplementary material for: TYMS promotes genomic instability and tumor progression in Ink4a/Arf null background
Source: Oncogene. 2023 Apr 27;42(23):1926–39. doi: 10.1038/s41388-023-02694-7 (PMC10244171; doi:10.1038/s41388-023-02694-7)

SUPPLEMENTARY INFORMATION

TYMS promotes genomic instability and tumor progression in *Ink4a/Arf* null background

Maria V. Guijarro, Akbar Nawab, Peter Dib, Sandra Burkett, Xiaoping Luo, Michael Feely, Elham Nasri, Robert P. Seifert, Frederic J. Kaye and Maria Zajac-Kaye

This file contains:

1. Supplementary Methods
2. Supplementary Figure legends
3. Supplementary Tables
4. Supplementary References
5. Supplementary Figures

SUPPLEMENTARY METHODS

Analysis of TYMS in TCGA human tumors

TYMS expression across human TCGA dataset compared to normal tissue was obtained and analyzed through http://ualcan.path.uab.edu.
TYMS and CDKN2A expression (TPM+1) data from frozen biopsies of B and T lymphomas was extracted from RNA-seq data from matrix GSE120124 [1] and data was plotted using GraphPad Prism 9 (GraphPad Software, USA).

Preparation of MEF cell cultures

Embryos from FVB mice were isolated at day 12.5 ad minced in trypsin for 20 minutes and then seeded in T75 flasks in DMEM supplemented with 10%FBS. Cells were split 1:3 when flask was confluent and passaged 2 times until homogeneous culture to be frozen or expanded for further studies.

Senescence associated β-Galactosidase (SA β-Gal) staining

In situ whole organ staining was performed on whole-mount tissues isolated from mice following sacrifice using the Senescence β-Galactosidase Staining Kit (Cell Signaling #9860). Briefly, tissues were fixed at room temperature for 2 h, in a solution containing 2% formaldehyde and 0.2% glutaraldehyde in PBS. Then, tissues were washed three times with PBS, and incubated overnight at 37°C with the Staining Solution containing X-gal in N-N-dimethylformamide (pH 6.0). Tissues were then dehydrated with 50% and 70% ethanol consecutively and embedded in paraffin for serial sectioning. Sections were counterstained with eosin.

For SA β-Gal staining of MEFS-V and MEFS-TS, cells were washed with PBS, fixed with 4% formaldehyde in PBS for 15 min and stained using the Senescence β-Galactosidase Staining Kit (Cell Signaling #9860). After an overnight incubation at 37°C, cells were analyzed for blue staining under a phase-contrast microscope. At least 11 field pictures were taken using a Leica Microscope with LAS AF Software X 3.7.4. Quantification was performed as a percentage of the total amount of cells per field.

Retroviral production

To generate retrovirus, $2x10^6$ HEK293T cells were seeded in a 10 cm plate and the following day, transfected with 3 µg of pLNCX-TS or pLNCX empty vector and the retroviral packaging plasmids

pMD-MuLV (1 µg) and pMD-G (1.5 µg) (Addgene) using X-tremeGENE 9 DNA Transfection Reagent (Roche #6365809001) as per the manufacturer's instructions. Briefly, the transfection mixture was prepared by mixing pLNCX-TS or pLNCX plasmids with the packaging plasmids and 10 µl of X-tremeGENE reagent into media up to 1 ml. After 15 minutes incubation, mixture was added dropwise to the cells. Supernatant was collected 48h after transfection and filtered through a 0.45 µm syringe filter.

TYMS overexpression in MEFS

For transduction, $0.1x10^6$ MEFS WT were seeded per well in 6 well plates. 2 ml of the supernatant was used per well containing 8 µg/ml of polybrene (Millipore) and centrifuged for 15 minutes, 1000 rpm at RT. After 24h, virus containing media was replaced with regular media and infected again the following day.

RNA extraction and RT-qPCR for Senescence-associated secretory phenotype genes

Total RNA was prepared from frozen mouse tissue using Trizol reagent (Invitrogen). cDNA was synthesized using High Capacity cDNA Reverse Transcription Kit (Applied Biosystems #4368814) following manufacturer's instructions. Briefly, 500 ng of total RNA was converted into cDNA and amplified by real-time PCR with gene-specific primers using SYBR Green PCR Master Mix (Applied Biosystems #4309155). Primers used are specified in the table below.

Target gene	Protein	Forward primer (5' > 3')	Reverse primer (5' > 3')
Il6	IL-6	TGATTGTATGAACAACGATGATGC	GGACTCTGGCTTTGTCTTTCTTGT
Cxcl1	CXCL1	CTGGGATTCACCTCAAGAACATC	CAGGGTCAAGGCAAGCCTC
Actb	Beta-Actin	CCTTCTTGGGTATGGAATCCTGT	CACTGTGTTGGCATAGAGGTCTTTAC

SUPPLEMENTARY FIGURE LEGENDS

Fig. S1 TYMS does not induce senescence in heterozygous or null *Ink4a/*Arf tumors
A RT-qPCR analysis showing no significant differences in the relative CXCL1 and IL6 mRNA expression comparing *Ink4a/Arf* $^{+/-}$ (*n* = 3) to *hTS/Ink4a/Arf* $^{+/-}$ (*n* = 5) mice. RNA was extracted from frozen tumors from mice included in Fig. 1B. Values represent mean ± SEM. *P* = 0.1613 for CXCL1 and *P* = 0.4772 for IL6, by two-tailed unpaired t-test. **B** RT-qPCR showing no significant differences in relative CXCL1 and IL6 mRNA levels from *Ink4a/Arf* $^{-/-}$ (*n* = 7) compared to *hTS/Ink4a/Arf* $^{-/-}$ (*n* = 10) frozen spleens infiltrated with lymphoma from animals included in Fig. 2. Values represent mean ± SEM. *P* = 0.7509 for CXCL1 and *P* = 0.1574 for IL6, by two-tailed unpaired t-test. **C** RT-qPCR showing no significant differences in relative CXCL1 and IL6 mRNA levels in *Ink4a/Arf* $^{-/-}$ (*n* = 2) compared to *hTS/Ink4a/Arf* $^{-/-}$ (*n* = 4) frozen fibrosarcomas from animals in Fig. 3. Values represent mean ± SEM. *P* = 0.3597 for CXCL1 and *P* = 0.3674 for IL6. by two-tailed unpaired t-test. **D** Senescence associated β-Galactosidase staining (SA β-Gal) freshly harvested liver, lung, spleen infiltrated with lymphoma and soft tissue sarcoma from K566 and K592 mice overexpressing TYMS in *Ink4a/Arf* $^{-/-}$ null background. No blue staining indicative of senescent cells was observed in any of the tissues. H&E and SA β-Gal staining are shown. Error bars represent 50 μM except in K566 spleen that represent 20 μM.

Fig. S2 TYMS does not induce senescence in WT MEFS
A Relative hTS mRNA levels of WT MEF infected with empty vector (MEF-V) compared to MEF overexpressing hTS (MEF-TS) after 8 days of infection. Data represents mean of 3 technical replicates ± SD. ***P* = 0.017 calculated by two-tailed unpaired t-test. **B** Percentage of senescent cells in MEF-TS compared to MEF-V from C and D. Pictures of 11 fields were taken and quantified. Values represent mean ± SD, *P* = 0.8974 by two-tailed unpaired t-test. **C** Representative RT-qPCR showing no significant differences in the relative CXCL1 mRNA levels in MEF from B. Values represent mean ± SD. *P* = 0.7578 by two-tailed unpaired t-test.

Fig. S3 IHC and flow cytometry marker expression in histiocytic sarcoma
A CD3 negative immunostaining in spleen and liver infiltrated with histiocytic sarcoma. Tissues belong to same animal (mouse ID 5219) as in Fig. 2A – a and i. Scale bar represents 50 μm. **B** CD11b positive splenic cells detected by flow cytometry. Spleen belongs to same animal shown in A. **C** Quantification of Mac-2, CD45R and CD3 expression by IHC from mice in Fig. 2; and CD11b by flow cytometry in animals from Figure 2A. HS: histiocytic sarcoma, Lymph: lymphoma;

(+) indicates presence and (–) indicates absence of expression.

Fig. S4 TYMS (TS) is highly expressed across TCGA human tumors

A Comparison of TS mRNA levels across TCGA tumors (expressed as log2 transcripts per million, TPM+1) compared to normal tissue evaluated using UALCAN portal [2]. BLCA: bladder urothelial carcinoma; BRCA: breast invasive carcinoma; CESC: cervical squamous cell carcinoma; CHOL: cholangiocarcinoma; COAD: colon adenocarcinoma; ESCA: esophageal carcinoma; GBM: glioblastoma multiforme; HNSC: head and neck squamous cell carcinoma; KICH: kidney chromophobe; KIRC: kidney renal clear cell carcinoma; KIRP: kidney renal papillary cell carcinoma; LIHC: liver hepatocellular carcinoma; LUAD: lung adenocarcinoma; LUSC: lung squamous cell carcinoma; PAAD: pancreatic adenocarcinoma; PRAD: prostate adenocarcinoma; PCPG: pheochromocytoma and paraganglioma; READ: rectum adenocarcinoma; SARC: sarcoma; SKCM: skin cutaneous melanoma; THCA: thyroid carcinoma; THYM: thymoma; STAD: stomach adenocarcinoma; UCEC: uterine corpus endometrial carcinoma. **B** TS is highly expressed in Diffuse Large B-cell Lymphoma. TS mRNA levels across TCGA tumors are expressed as log2 transcripts per million (TPM+1) evaluated using the UALCAN portal. ACC: adrenocortical carcinoma; DLBLC: diffuse large B-cell lymphoma; LGG: brain lower grade glioma; MESO: mesothelioma; OV: ovarian serous cystadenocarcinoma; LAML: acute myeloid leukemia; TGCT: testicular germ cell tumor; THYM: thymoma; UCS: uterine carcinosarcoma; UVM: uveal melanoma. **C** Non-Hodgkin lymphoma datamining from public dataset (see Material and Methods) show high levels of TS. ABC DLBCL, B-cell diffuse large B-cell lymphoma; BL: Burkitt lymphoma; FL: follicular lymphoma; GCB DLBCL: germinal center B-cell diffuse large B-cell lymphoma; ML: mantle cell lymphoma; PTCL: peripheral T-cell lymphoma; ALCL: anaplastic large cell lymphoma. **D** Non-Hodgkin lymphoma dataset from C show high TS levels and low CDKN2A expression. Heatmap of mRNA levels expressed in log 2 transcripts per million, TPM+1.

SUPPLEMENTARY TABLES

Table S1 Number of distinct tumors per mouse and STS developed simultaneously with other tumors.

	Ink4a/Arf $^{-/-}$		*hTS/Ink4a/Arf* $^{-/-}$	
	Cases/total mice	%	Cases/total mice	%
Number of distinct tumors per mouse				
1	46/52	88.5	66/84	78.5
2	6/52	11.5	16/84	19
3	0/52	0	2/84	2.4
Sarcoma (non-fibrosarcoma) plus another tumor:				
Lymphoma and Fibrosarcoma	0	0	1/5	20
Histiocytic sarcoma	0	0	3/5	60
Fibrosarcoma plus another tumor:				
Lymphoma	2/10	20	6/16	37.5
Histiocytic sarcoma	1/10	10	1/16	6.25

Table S2 SKY analysis of chromosomal abnormalities in cells derived from mouse histiocytic sarcoma

	Cell lines	# of cells	Karyotype	Aneuploidy Loss	Aneuploidy Gain	Translocation
Ink4a/Arf $^{-/-}$	5318	1	39, XX, -1	✓		
		1	39, XX, -6	✓		
		1	39, X	✓		
		2	37, XX, -15, -16, -17	✓		
		19	40, XX			
	Total	24		4<br>16.7%	0<br>0%	0<br>0%
hTS/Ink4a/Arf $^{-/-}$	5278	1	37, X, -Y, -13, -17	✓		
		1	38, -X, -Y	✓		
		1	38, XY, -10, -14, T(4;13), T(Y;16)	✓		✓
		1	39, X, -Y	✓		
		1	39, XY, -19	✓		
		1	37, X, -Y, -1, -2, -6, +8	✓	✓	
		1	38, XY, -6, -6, +8, -9	✓	✓	
		1	39, -XY, +8	✓	✓	
		1	39, XY, -1, +8	✓	✓	
		1	39, XY, +8, -18	✓	✓	
		1	39, XY, +8, -12, -17	✓	✓	
		1	39, XY, -1, +8, Del(11),-13	✓	✓	
		1	39, XY, -4, +8, -10, T(18;15), T(2;19)	✓	✓	✓
		9	41, XY, +8		✓	
		1	41, XY, +Del(8)		✓	
		6	40, XY			
	Total	29		13<br>44.8%	18<br>62.1%	2<br>6.9%

REFERENCES

1 Pericart S, Tosolini M, Gravelle P, Rossi C, Traverse-Glehen A, Amara N *et al*. Profiling Immune Escape in Hodgkin's and Diffuse large B-Cell Lymphomas Using the Transcriptome and Immunostaining. *Cancers (Basel)* 2018; 10: 414.

Supplemental Figure S1

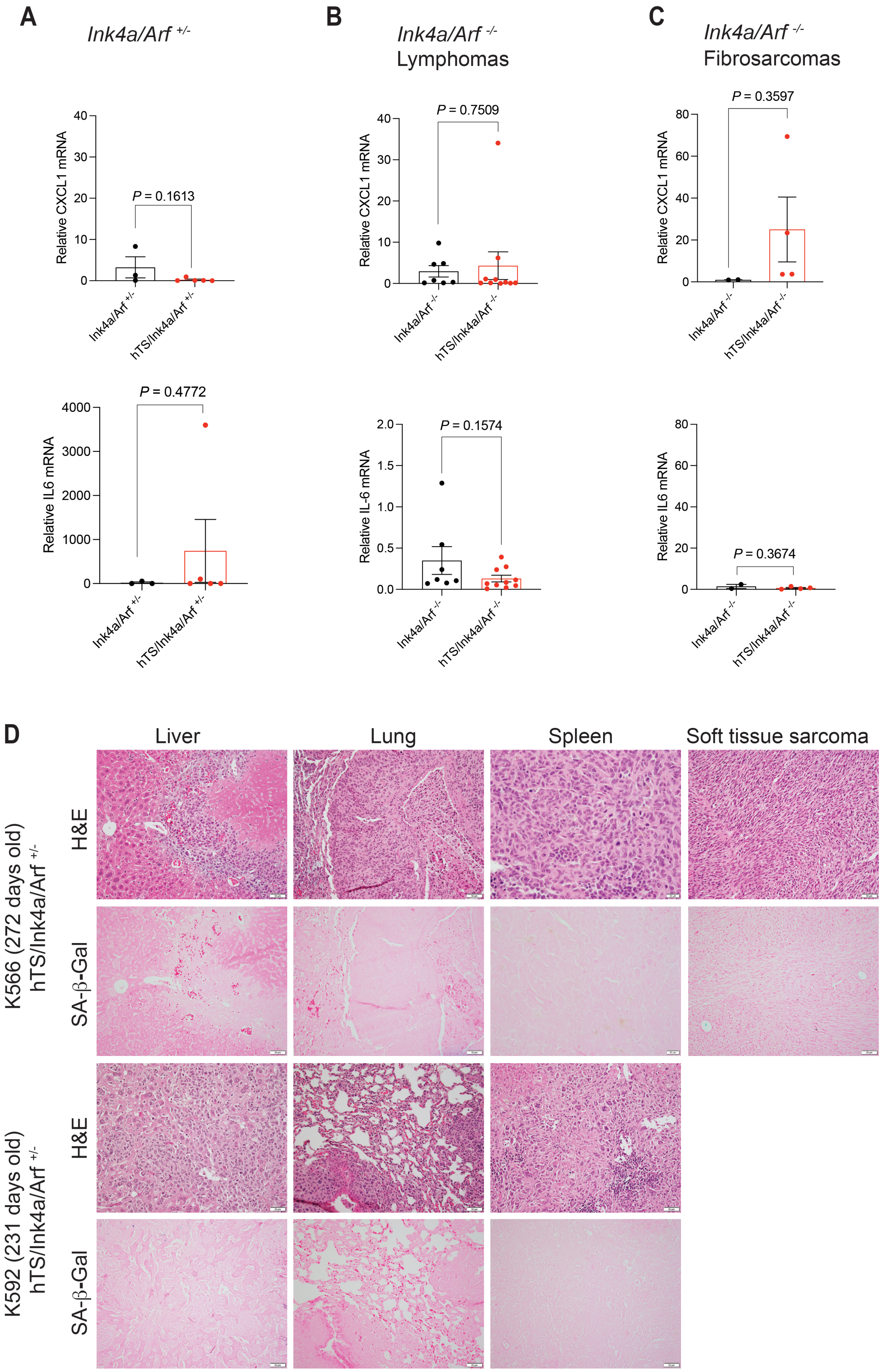

Supplemental Figure S2

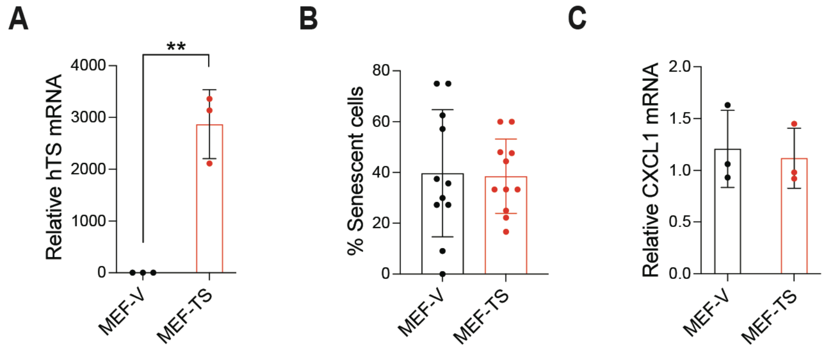

Supplemental Figure S3

A

CD11b

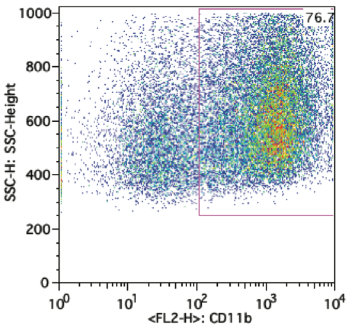

B

CD3

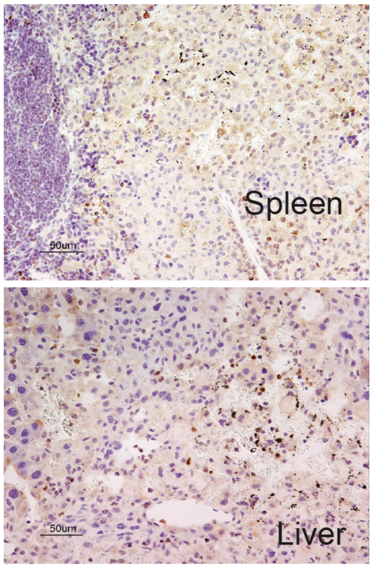

C

Mouse ID	Organ	Pathology	IHC			Flow cytometry (spleen)
			HS-Mac2	Lymph-CD45R	T Lymph-CD3	HS-CD11b
5219	Spleen	HS + L	+	+	-	76.7%
5219	Liver	HS	+	-	-	
5213	Lymph Nodes	HS + L	+	+	+	44.3%
5242	Kidney	HS	+	-	-	70.8%
5172	Pancreas	HS	+	-	-	68.6%

Supplemental Figure S4

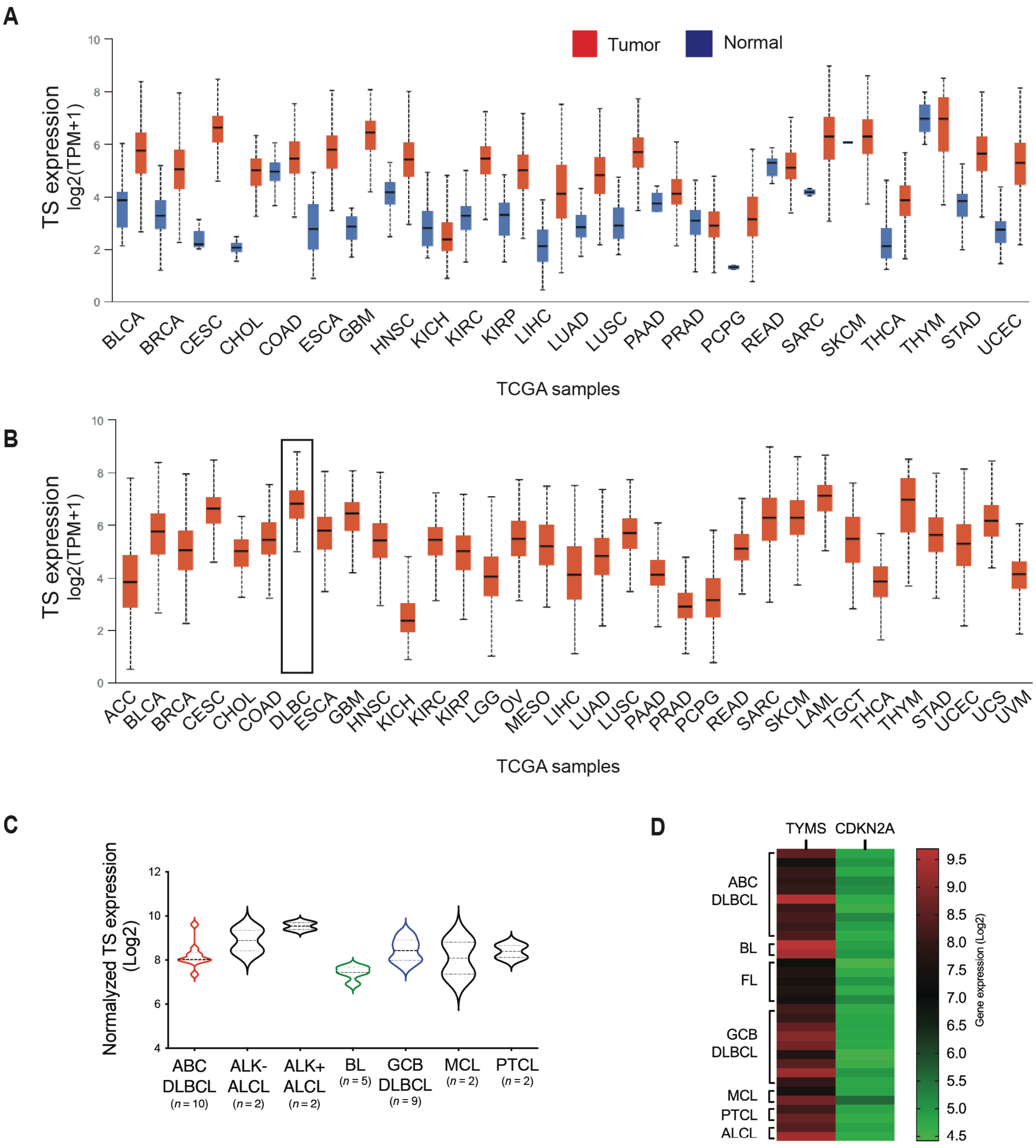

Supplement: Supplementary file 1 — Supplemental Material [file 41388_2023_2694_MOESM1_ESM.pdf]
